# Saccadic Eye Movement in Mild Cognitive Impairment and Alzheimer’s Disease: A Systematic Review and Meta-Analysis

**DOI:** 10.1007/s11065-021-09495-3

**Published:** 2021-05-06

**Authors:** Julius Opwonya, Dieu Ni Thi Doan, Seul Gee Kim, Joong Il Kim, Boncho Ku, Soochan Kim, Sunju Park, Jaeuk U. Kim

**Affiliations:** 1 Future Medicine Division, Korea Institute of Oriental Medicine, Daejeon, Republic of Korea; 2 Department of Preventive Medicine, College of Korean Medicine, Daejeon University, Daejeon, Republic of Korea; 3 Korean Convergence Medicine, University of Science and Technology, Daejeon, Republic of Korea; 4 Department of Electrical and Electronic Engineering, Hankyong National University, Anseong, Republic of Korea

**Keywords:** Dementia, Alzheimer’s dementia, Eye movements, Saccades, Gap effect, Anti-effect

## Abstract

Alzheimer’s disease (AD) is the leading cause of dementia, and mild cognitive impairment (MCI) is considered the transitional state to AD dementia (ADD) and other types of dementia, whose symptoms are accompanied by altered eye movement. In this work, we reviewed the existing literature and conducted a meta-analysis to extract relevant eye movement parameters that are significantly altered owing to ADD and MCI. We conducted a systematic review of 35 eligible original publications in saccade paradigms and a meta-analysis of 27 articles with specified task conditions, which used mainly gap and overlap conditions in both prosaccade and antisaccade paradigms. The meta-analysis revealed that prosaccade and antisaccade latencies and frequency of antisaccade errors showed significant alterations for both MCI and ADD. First, both prosaccade and antisaccade paradigms differentiated patients with ADD and MCI from controls, however, antisaccade paradigms was more effective than prosaccade paradigms in distinguishing patients from controls. Second, during prosaccade in the gap and overlap conditions, patients with ADD had significantly longer latencies than patients with MCI, and the trend was similar during antisaccade in the gap condition as patients with ADD had significantly more errors than patients with MCI. The anti-effect magnitude was similar between controls and patients, and the magnitude of the latency of the gap effect varied among healthy controls and MCI and ADD subjects, but the effect size of the latency remained large in both patients. These findings suggest that, using gap effect, anti-effect, and specific choices of saccade paradigms and conditions, distinctions could be made between MCI and ADD patients as well as between patients and controls.

**Supplementary Information:**

The online version contains supplementary material available at 10.1007/s11065-021-09495-3.

## Introduction

Life expectancy is increasing rapidly for a number of reasons, such as better health care and hygiene, healthier lifestyles, improved food security, and lower child mortality rates (World Health Organization, 2020). We now live longer and healthier lives than our ancestors just a few generations ago. Nevertheless, this dramatic increase in life expectancy has not been accompanied by a proportionate increase in quality of life, particularly for the elderly, who suffer from numerous age-related conditions. Rather, the increase in longevity has increased the risk of disease, disability, dementia, and advanced aging prior to death (Kassebaum et al., 2016).

The term “dementia” is generally understood as a behavioral or cognitive decline sufficiently serious to affect the capacity of a person to undertake everyday tasks but not associated with psychiatric disorders (G. M. McKhann et al., 2011). Dementia due to Alzheimer’s disease (ADD) accounts for an estimated 60 to 80 percent of dementia cases (Association, 2019) and has overtaken cancer as the most feared disease according to a recent survey (Alzheimer’s Disease International 2018, September). ADD is marked by a gradual cognitive decline occurring continuously over a long period, and it is understood to start two decades or more before symptoms emerge (Association 2019; Monsell et al., 2014; Resnick et al., 2010; Savonenko et al., 2015; Wilson et al., 2010). ADD is well known to impact various cognitive processes, with substantial episodic amnesia from the initial stages of the disease as well as deterioration in semantic memory, language, inhibitory control, attention, visuospatial function, and executive dysfunction (Bondi et al., 2017; Chau et al., 2015; Crawford et al., 2015b; Crawford & Higham, 2016; Hellmuth et al., 2012; T. J. Shakespeare et al., 2015; Whitehead et al., 2018).

Present research has identified three stages of Alzheimer’s disease (AD): preclinical AD, mild cognitive impairment (MCI) due to AD, and ADD (Association 2019; Jack et al., 2018). Preclinical AD spans from the first neuropathologic brain lesions to the onset of the first clinical symptoms of AD (Bruno Dubois et al., 2016). MCI is marked by cognitive deterioration greater than anticipated for the individual's age and level of education, although this does not significantly disrupt everyday life activities (Gauthier et al., 2006; G. M. McKhann et al., 2011). MCI can be categorized based on clinical presentation as amnestic MCI (aMCI) and nonamnestic MCI (naMCI), or the number of cognitive domains affected as single cognitive domain or multiple cognitive domains (Roberts & Knopman, 2013). The number of affected domains has important implications for understanding the extent of the underlying brain disease or pathology, disease severity, and likelihood of progression to dementia. MCI with primarily memory deficits is called as amnestic MCI. naMCI includes MCI with problems in thinking skills, inability to make sound decisions and judgments, and inability to take the sequential steps needed to perform relatively complex tasks (Khan, 2016). Typically, patients with MCI convert to ADD at an average of nearly 15% annually, although this prevalence rate varies considerably due to the various MCI diagnostic methods (Libon et al., 2014; Mitchell & Shiri-Feshki, 2009). In general, individuals with aMCI eventually develop into ADD and those with naMCI develop into non-AD dementias (Gauthier et al., 2006; Khan, 2016). Overall, MCI may be temporary, persistent, or progress to other types of neurodegenerative diseases such as AD dementia (Mitchell & Shiri-Feshki, 2009).

With the rise of the aging population, we expect to see a rise in the number of individuals afflicted by ADD. Current solutions for treatment are ineffective, as a number of AD drugs have been tested, but no currently approved drugs can cure the disease, all drugs on the market provide only symptomatic relief. Additionally, the diagnosis of ADD relies largely on documenting cognitive decline, by which time the disease has already caused severe brain damage (G. M. McKhann et al., 2011). For this reason, there is a need for early diagnosis in order to delay or prevent the onset of symptoms (Cummings et al., 2019).

Various approaches, such as genetic testing, biological markers, and structural and functional neuroimaging, have been proposed to improve screening and timely identification of cognitive decline. Among them, biological markers may offer the most promising path to the discovery of an easy and accurate way to detect MCI and ADD before symptoms begin (Jack et al., 2018). Several potential AD biomarkers are being studied to assess their ability to detect prodromal AD and offer objective, dependable measures of disease progress (Goldman & Van Deerlin, 2018). A well-known biomarker used to evaluate the risk or presence of AD is amyloid beta, which is detectable in cerebrospinal fluid (CSF) and blood plasma (Jack et al., 2018; Nakamura et al., 2018). Other indicators of early AD include cortical and subcortical alterations, destruction in the limbic area, cerebral cortex, hippocampus and subcortical nuclei, and eye function changes (Braak & Braak, 1995; Daffner et al., 1992; Katz & Rimmer, 1989). The current biomarkers used in AD studies are either expensive or invasive, hence, we believe that for widespread use, the development of affordable or noninvasive biomarkers for screening or monitoring neuropathological changes is required.

Eye tracking (ET) technology is becoming popular due to the development of accurate, affordable, moveable and easy-to-use eye trackers. ET can be employed in various environments, enabling research of various population groups (Bueno et al., 2019). The eye shares many neural and vascular similarities to the brain and numerous cortical and subcortical regions, which are affected by AD and participate in the triggering and regulation of eye movements (EMs). Consequently, ET can provide an indirect link to neuronal and cognitive functioning (Broerse et al., 2001; Holmqvist et al., 2011; Jamadar et al., 2013; McDowell et al., 2008). Thus, ET may offer a method for monitoring of preclinical, MCI, and ADD stages in a way that is potentially sensitive to the cognitive disease process.

ET metrics might be applied to different aspects of oculomotor behavior such as fixations, smooth pursuit, vergence, vestibular-ocular movements, optokinetic movements, saccades, and pupil responses (Borys & Plechawska-Wójcik, 2017; Duchowski, 2007). Fixations maintain the eye steady during purposeful gaze when the head is stationary. Smooth pursuit movements hold the image of a mobile target on the fovea centralis. Vergence movements shift the eyes in a reverse course to facilitate image positioning on both foveae. Vestibular-ocular reflexes maintain images on the retina during quick motions of the head. Saccades swiftly shift the fovea to a new focus (Mack et al., 2013). Pupil responses (dilation and constriction) are a physiological response that varies the size of the pupil. To date, fixation, smooth pursuit, and saccades are the most common components in EMs assessed in ET tasks for AD (Daffner et al., 1992; Garbutt et al., 2008; Pavisic et al., 2017).

EMs and pupillary responses offer accurate information regarding executive function that can be assessed by oculometrics such as saccade amplitude, saccade latency, saccade peak velocity, fixation duration, latency to pupil constriction, peak pupil constriction, baseline pupil diameter and other measures that are presumed to reflect neural mechanisms of goal-directed behavior, decision making, learning, memory, and attention (Borys & Plechawska-Wójcik, 2017; Eckstein et al., 2017; Holmqvist et al., 2011; Hutton 2008; Luna et al., 2008; Marandi & Gazerani, 2019). These unique characteristics make the eye a relatively inexpensive biomarker for cognitive evaluation and the evolution of AD, which carries the potential for wide implementation (Anderson & MacAskill, 2013; Molitor et al., 2015).

**Fig. 1:**
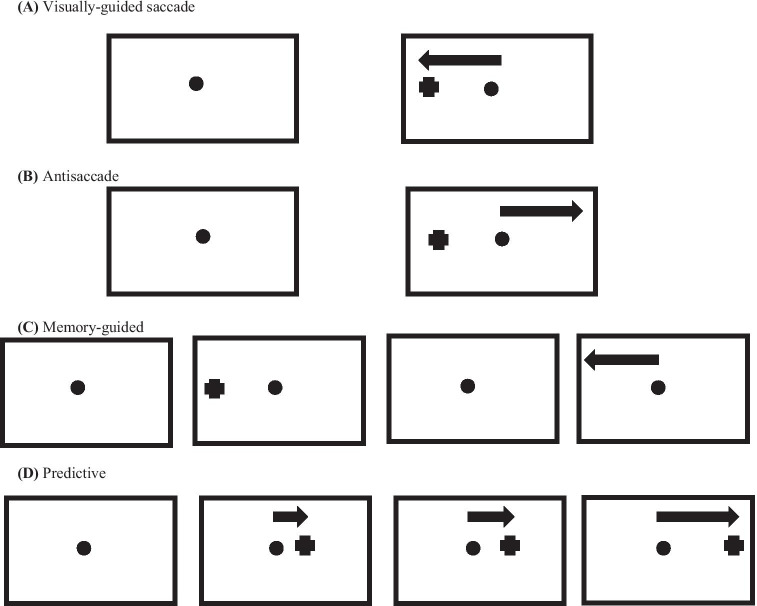
Saccadic paradigms. (**A**) Visually guided saccade: a visual stimulus is shown randomly to the right or left side of a central point of fixation and participants are directed to react with quick and accurate EMs. (**B**) Antisaccade: the EMs are oriented toward a spatial position in the visual field contrasting the stimulus. (**C**) Memory-guided saccade: participants are directed to inhibit natural reflexive EMs when a new stimulus appears as well as to suppress the saccade until the central fixation point is offset. At the time of the saccadic initiation, there is no visual information on the location of the previously displayed target. (**D**) Predictive saccade: a visible target steps in spatial variants in a foreseeable chronological sequence

ET dependent evaluation of EMs, in particular examination of saccade properties, is especially helpful in assessing the stage of disease in patients with mild motor function disorders and cognitive impairments, such as ADD (Anderson & MacAskill, 2013). In addition, laboratory-based ET, especially testing of saccade properties, can provide relevant information regarding progression or reversion in neurodegenerative diseases (Anderson & MacAskill, 2013). Two main categories of saccadic EMs can be differentiated: visually guided saccades (also known as reflexive, refixation, or prosaccades) and voluntary (or volitional) saccades. A visually guided saccade can be described as an involuntary positioning reaction to a new event in the field of vision, whereas voluntary saccades result from purposeful activity in a variety of paradigms such as antisaccades, memory-guided saccades or predictive saccades. In antisaccades, the gaze is oriented to the opposite location of the peripheral target onset (Hallett, 1978). In memory-guided saccades, subjects fixate on a central stimulus, and a peripheral focus is shown momentarily, signaling the position for a corresponding saccade, then they conduct saccadic EM toward the target stimulus. In predictive saccades, participants typically direct their gaze in expectation of the emergence of a target in a specific spot with a fixed temporal frequency (Broerse et al., 2001) (Fig. 1).

**Fig. 2:**
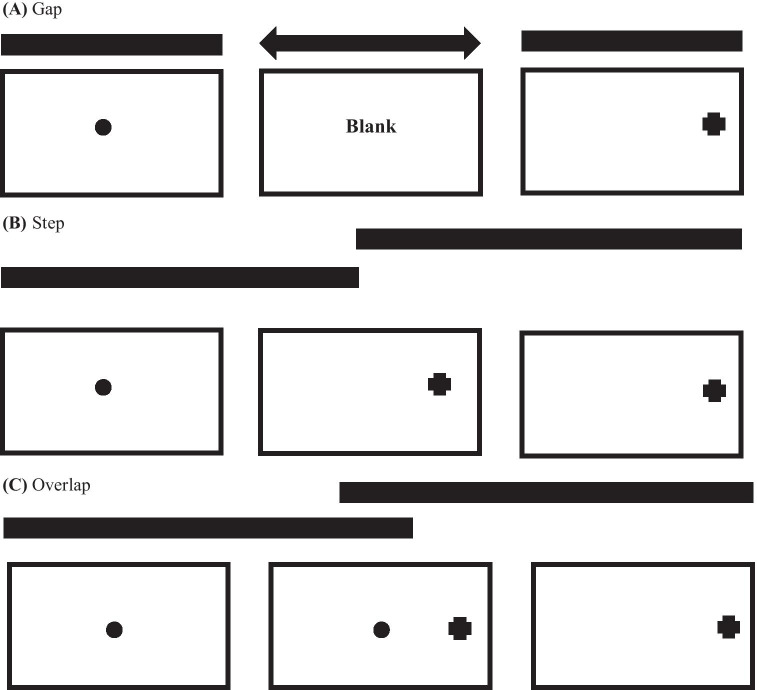
Elementary trial technique for saccade paradigms, showing (**A**) gap, (**B**) step and (**C**) overlap conditions

Within these paradigms, many conditions are possible. Among the most popular conditions used in saccade tasks relates the timing between the central fixation stimulus offset and the appearance of the peripheral stimulus target. In standardized ‘‘step” trials, the central fixation offset matches up with the peripheral target appearance. In “gap” trials, the central fixation offset leads the peripheral target appearance, whereas in “overlap” trials, the central fixation stimulus is noticeable after peripheral target appearance (Hutton, 2008: see Fig. 2).

The gap effect refers to the shorter saccade latency in gap trials than in other conditions. The effect is due to a variety of possible mechanisms that are structured to facilitate in the maintenance of fixation (Pratt et al., 2006). One explanation is that the absence of the fixation point in the gap trials allows attention to be detached until the target emerges, leading to a faster saccade latency, which are referred to as express saccades (Fischer & Ramsperger, 1984), while visual attention is engaged during overlap trials and saccades are suppressed, leading to slower latencies (Fischer et al., 1993; Fischer & Breitmeyer, 1987; Fischer & Ramsperger, 1984). Some researchers have construed the offset of the fixation stimulus in the gap task to serve as an alerting signal, leading to a reduction in saccade latencies (Reuter-Lorenz et al., 1995). Overall, the gap effect appears to reflect both attentional disengagement ‘‘fixation release” and warning components. This fixation release aspect has been suggested to be regulated by low-level neural connections in the superior colliculus (Reuter-Lorenz et al., 1991). According to this description, the removal of the central stimulus results to diminished activity of the fixation neuron in the superior colliculus, thereby disinhibiting movement cells and aiding the beginning of a successive saccade (Hutton, 2008).

The anti-effect refers to a decrease in the latency of visually guided saccade trials relative to antisaccade trials (Hallett, 1978; Hallett & Adams, 1980; Douglas P. Munoz & Everling, 2004), which may be attributed to the additional cognitive processes in the antisaccade trials. The areas of the brain controlling saccadic EMs have been established from preclinical and clinical lesion and neuroimaging studies (McDowell et al., 2008; Pierrot-Deseilligny et al., 2004). The generation of basic visually guided saccades and more sophisticated voluntary saccades involves similar core neural connections, with additional brain areas supporting the relevant cognitive functions (McDowell et al., 2008). Both types of saccades have recognizable neural pathways directly linked to their respective cognitive processes (Broerse et al., 2001; McDowell et al., 2008). Sensory-motor programming in a visually guided paradigm may be guided by different cortical and subcortical networks contingent on the nature of the saccadic paradigm. The network involved in visually guided saccade generation includes striatum, thalamus, superior colliculus, and cerebellar vermis subcortical regions as well as frontal, occipital, and parietal cortical regions. This involves the incorporation of spatial attention, visual processing and a specifically focused motor system but limits requirements on higher-order executive functions. A wide variety of higher-order processes for example attention and knowledge acquisition have been found to influence performance on visually guided saccades (Hutton, 2008).

In volitional saccades, there is a greater demand on higher-level executive control leading to an increasingly complex patterns of brain stimulation. In antisaccade trials, at least 2 different steps are necessary compared to visually guided trials: the inhibition of the reflexive response to make a visually guided saccade to the target and the reversal of the stimulus location into a voluntary motor command to look the other way from the stimulus. Antisaccade execution incorporates a fronto-parieto-subcortical network, comprising dorsolateral prefrontal cortex (DLPFC), supplementary eye field (SEF), frontal eye fields (FEFs), anterior cingulate cortex (ACC), posterior parietal cortex, thalamus, and striatum (Hutton & Ettinger, 2006). Broadly, antisaccade trials activate the oculomotor network more than visually guided trials and may also recruit extra brain areas such as DLPFC and ACC, which are unnecessary in visually guided trials. Activity in these areas is additionally noted during voluntary saccades (such as memory-guided saccades, antisaccades, and predictive saccades); each of them need sophisticated executive processes. These extra demands are facilitated by changes in saccade circuitry activity and by recruitment of extra brain areas. The antisaccade task encompasses a wide range of cognitive processes, such as decision making, working memory, goal-oriented behavior, knowledge acquisition, and attention (Jamadar et al., 2013). Visual cortical activity is regulated as a function of the task requirements and can predict the kind of saccade to be generated, likely through a top-down control process (Broerse et al., 2001; McDowell et al., 2008)

New research utilizing saccadic paradigms has provided evidence of precise abnormalities strongly associated with cognitive measures using conventional neuropsychological tests (Crawford & Higham, 2016; Crawford et al., 2013; Lagun et al., 2011). Several studies have found that EMs between patients with MCI and those with ADD are different from those of healthy age-matched controls (Boxer et al., 2006; Chehrehnegar et al., 2019; Garbutt et al., 2008; Heuer et al., 2013; Holden et al., 2018; Peltsch et al., 2014; Yang et al., 2011, 2013). However, there is still considerable ambiguity in choosing parameters that are relevant in distinguishing between controls and patients with AD. The disparity in saccade paradigm formats may account for the substantial part of the variance seen across studies; hence, the assessment of methodological approaches is of particular importance. In addition, the magnitude and significance of longer reaction times on antisaccade trials than on visually guided trials (the anti-effect), the gap effect, and antisaccade task measures such as antisaccade latency, latency of incorrect prosaccades, numerous spatial accuracy measures, such as the amplitude of correct and incorrect saccades and the final eye position of correct responses, and errors (prosaccades toward the target that are not corrected), which have been found to vary in healthy humans, vary considerably across studies and laboratories, with some studies reporting rates as low as 5% and others as high as 25% (Hutton & Ettinger, 2006). Furthermore, the time to correct errors (the time between an incorrect prosaccade and subsequent corrective antisaccade) in patients with MCI and patients with ADD has not been dealt with in depth.

A recent meta-analytic review of the literature on visually guided and volitional saccade paradigms found that patients with ADD but not patients with MCI had longer visually guided latencies than controls. Additionally, for the volitional antisaccade task, antisaccade latencies did not differentiate between patient groups from healthy controls, but the frequency of antisaccade errors was significantly increased among patient groups compared with controls (Kahana Levy et al., 2018). One of the main limitations of the review was that they used saccade latency only in the gap condition for calculating effect sizes, and saccadic latency and error rates in other formats, such as step and overlap, were not explored. Consequently, this raises questions about the significance and relevance of other conditions not explored, such as overlap or step conditions, for distinguishing between patients with AD and controls.

As a step forward in improving the clinical usability of the EM technique, we review existing original articles and conduct meta-analyses to differentiate performances in saccadic EM of patients with MCI and patients with ADD from their normal controls based on various saccadic paradigms (e.g., visually guided vs. antisaccade paradigm) and on diverse conditions (e.g., gap, overlap, or step conditions).

## Methods for Systematic Review and Meta-Analysis

### Protocol and Registration

This systematic review has been registered with the International Prospective Register of Systematic Reviews (University of York Centre for Reviews and Dissemination, 2020); registration no. CRD42019138926; available from https://www.crd.york.ac.uk/prospero/display_record.php?ID=CRD42019138926) and is guided by the Preferred Reporting Items for Systematic Reviews and Meta-Analyses (PRISMA) statement (Liberati et al., 2009; Moher et al., 2009).

### Eligibility Criteria

We considered the following study designs: nonrandomized and randomized controlled trials (RCTs) and observational study designs such as cohort studies, cross-sectional studies and case–control studies, which investigated saccadic EMs in patients with MCI and patients with ADD in comparison with a healthy age-matched control group. The diagnosis of MCI (caused by any etiology) was based on the specific criteria as follows: Petersen criteria (Petersen et al., 1999), revised Petersen criteria (Petersen, 2004; Petersen et al., 2001), Winblad criteria (Winblad et al., 2004), Matthews criteria (Matthews et al., 2008), revised Matthews criteria for MCI (Artero et al., 2006), Clinical Dementia Rating (CDR) = 0.5 (Morris, 1993), the National Institute on Aging-Alzheimer's Association (NIA-AA) core clinical criteria (Albert et al., 2011), or a combination. For ADD, we used the following criteria: National Institute of Neurological and Communicative Disorders and Stroke–Alzheimer’s Disease and Related Disorders Association (NINCDS-ADRDA) criteria (G. McKhann et al., 1984; G. M. McKhann et al., 2011), DSM III (American Psychiatric, 1986) and DSM-IV (*Diagnostic and statistical manual of mental disorders : DSM-IV* 1994), DSM-IV-TR (Diagnostic criteria from DSM-IV-TR 2000), International Statistical Classification of Diseases and Related Health Problems ICD-10 (International statistical classification of diseases and related health problems, 2004)**,** and Dubois criteria (B. Dubois et al., 2007, 2010) or a combination. To be included in this review, articles had to be published in a peer-reviewed journal published between January 1980 and July 2020 and written in English. When several articles were published from the same parent study or dataset, only one article was included in the analysis based on the completeness of information that could be obtained from each article. All other articles published from shared datasets were excluded for reasons of non-independence, as they could potentially bias results (Liberati et al., 2009; von Elm et al., 2004). Finally, studies were excluded if they did not have an appropriate control group (e.g., children <18 years), participants were individuals with MCI or ADD not diagnosed according to specific criteria, or insufficient data were provided to calculate or estimate effect sizes and attempts to contact corresponding study authors were unsuccessful.

### Information Sources

We searched for published articles indexed in MEDLINE, EMBASE, and CENTRAL databases. A manual search of references and forward citations of relevant systematic reviews and relevant original research articles was also carried out to ensure that all potential studies were captured. The searches were concluded by July 30, 2020.

### Search Strategy

The search strategy was developed through a review of published literature and in consultation with a reviewer experienced in systematic reviews and adapted to other databases. The MEDLINE, EMBASE, and CENTRAL database search strategies are presented in Tables S1-S3 in the Online Resource.

### Study Selection

**Fig. 3:**
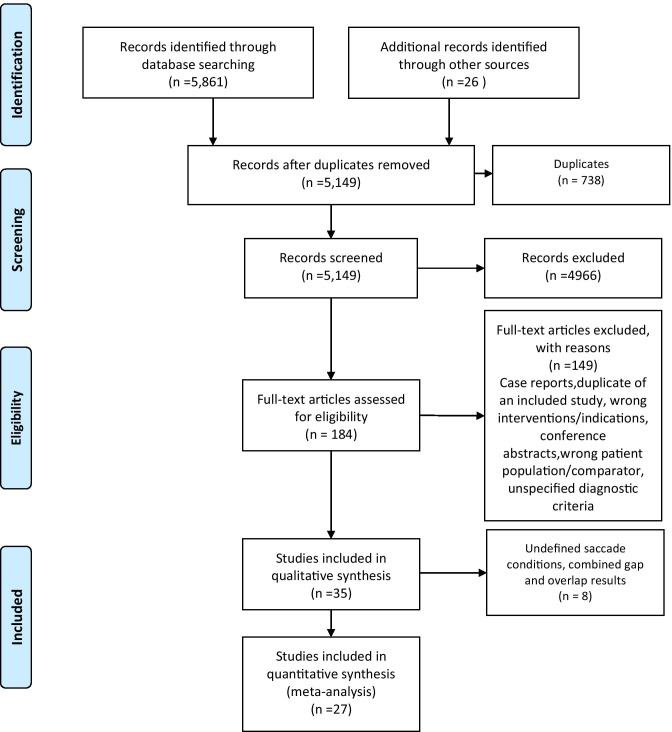
Flow diagram according to Preferred Reporting Items for Systematic Reviews and Meta-Analyses

All the identified articles were initially imported into Endnote (Ver. X9, Thomson Reuters, USA), and duplicate records were removed. These articles were then uploaded to Covidence systematic review software (The Cochrane Collaboration, 2020, July 22) where OJ and DDN screened the titles and abstracts. The reviewers independently screened the identified papers for inclusion using the registered protocol and made decisions about inclusion according to the eligibility criteria. Corresponding authors were contacted when the information in the published article was insufficient to decide eligibility. Disagreements were resolved by consensus or a third reviewer (KJU). Only those records that were included by both reviewers passed on to the final review stage. Reference lists of these eligible studies were manually checked to ensure that no potentially relevant articles were missed. The full texts of all papers not excluded based on title or abstract were screened. The number of articles included and excluded at the distinct phases was recorded as recommended and presented in a PRISMA flowchart (Moher et al., 2009: see Fig. 3).

### Data Extraction

Data were extracted independently by two reviewers (OJ and DDN) using a Microsoft Excel (2016) spreadsheet form tailored to the requirements of this systematic review. Disagreements were resolved through discussion with a third reviewer (KJU). If numerical data were missing from the results section, the reviewers extracted data with WebPlot Digitizer Version 4.2. Five study authors were contacted about missing data that were necessary to calculate effect sizes, and follow-up emails were initiated within one month when no response to the first emails was received. Two of these authors responded and provided the necessary information.

### Data Items

The extracted data included the title of article, first author, country, study design, demographic information of the sample (i.e., age in years,% male, education in years), cognitive status diagnostic criteria, scores on standard assessments of cognitive status (i.e., Mini-Mental State Examination, Montreal Cognitive Assessment), study population (e.g., MCI, ADD) and sample size per group, ET device and technique, oculomotor paradigm, saccade task condition (e.g., gap, step, overlap), saccade parameters (e.g., mean latency, amplitude, gain, errors, omissions, and anticipations), main findings and conclusions (Table 1). We used additional calculations such as, calculating the standard deviation (SD) from the standard error (SE) and sample size, standard errors from confidence intervals (CIs) and p values; absolute (difference) measures and standard errors from confidence intervals and p values; and ratio measures to obtain summary statistics where necessary.

### Risk of Bias in Individual Studies

The Risk of Bias in Non-Randomized Studies of Interventions (ROBINS-I) assessment tool was used since the study aimed to evaluate the efficacy of using various conditions during saccade-based EM as a screening, diagnostic, or monitoring method for patients with MCI and patients with ADD (Sterne et al., 2016). This tool includes seven specific bias domains, preintervention and postintervention. The domains are (1) confounding, (2) selection of participants, (3) classification of intervention, (4) deviation from interventions, (5) missing outcome data, (6) measurement of outcomes, and (7) selection of reported result overall. Risk of bias was rated as 0 - no information, 1 - low risk, 2 - moderate risk, 3 - serious risk, and 4 - critical risk. Two authors (OJ and DDN) independently assessed the risk of bias of the included articles. Disagreements were managed by consensus.

### Summary Measures

Effect sizes were shown in terms of standardized mean differences using Hedges’ g (unbiased), which includes a correction for small sample bias given the demonstrated tendency for studies with relatively small sample sizes to overestimate the true population effect (Hedges, 2016; Hedges & Olkin, 2014). For comparison, we also reported the difference in means (referred to as mean difference: MD) which is given by $\mathrm{MD}=\mathrm{M}1-\mathrm{M}2.$ There are several popular formulations of the standardized mean difference (SMD). The one implemented in RevMan is Hedges’ adjusted g, which is very similar to Cohen's d, but includes an adjustment for small sample bias. The formula for Hedges’ g = $\frac{\mathrm{M}1-\mathrm{M}2}{{\mathrm{SD}}_{\mathrm{pooled}}}\left(1-\frac{3}{4\mathrm{N}-9}\right)$, where M1 is the mean response for the patient group, M2 is the mean response for the control group, and N is the overall sample size including both patient and control groups (Hedges & Olkin, 2014). The pooled SD is calculated as SD_pooled_ = $\sqrt{\frac{\left(\left(\mathrm{N}1-1\right){\mathrm{SD}1}^{2}\right)+\left(\left(\mathrm{N}2-1\right){\mathrm{SD}2}^{2}\right)}{(\mathrm{N}1+\mathrm{N}2)-2}}$, 

where N1 is the patient group sample size, N2 is the control group sample size, SD1 is the SD of the mean for the patient group, and SD2 is the SD of the mean for the control group. All effects were calculated such that a positive effect size corresponds to longer latency or higher frequency of errors during visually guided and antisaccades tasks in the patient groups (MCI and ADD) than in the control group.

### Synthesis of Results

A random-effects model was assumed given that heterogeneity in effect sizes was expected to exceed that which could be explained by sampling error alone (Deeks JJ, 2019; Rothstein et al., 2013). To address the primary aim of this review, the results from different saccade paradigms were pooled according to condition (gap, step, and overlap) to determine an overall mean effect size (Hedges & Olkin, 2014). Macros available in Review Manager Version 5.3 software (Cochrane, London, UK) and JASP computer software, version 0.13.1 were used to aggregate a mean effect size and 95% CI.

Heterogeneity of effect sizes was identified using Chi^2^ (χ^2^, or chi-square, Q) and quantified using the I^2^ statistic (*Cochrane handbook for systematic reviews of interventions* 2020). Chi^2^ is calculated as the weighted sum of squared deviations of each study’s effect size from the overall mean effect size and provides significance test for heterogeneity (Borenstein et al., 2011). I^2^ describes the percentage of the variability in effect estimates that is due to heterogeneity rather than sampling error (chance) (*Cochrane handbook for systematic reviews of interventions,* 2020). The formula for I^2^ $=\left(\frac{Q-\mathrm{df}}{Q}\right)\mathrm{x }100\mathrm{\%}$, 

where Q is the Chi^2^ statistic and df is its degrees of freedom (Higgins & Thompson, 2002; Higgins et al., 2003). In the meta-analysis, I^2^ values of 25%, 50%, and 75% represented low, moderate, and high degrees of heterogeneity, respectively (Higgins et al., 2003). However, it is important to note that I^2^ is a measure of relative heterogeneity, and a high I^2^ may be observed in the context of smaller absolute heterogeneity. Thus, Tau^2^ (Tau-squared, τ^2^) was also calculated to incorporate a measure of the extent of variation, or heterogeneity, among the intervention effects observed in the different studies. Τau^2^ is defined as the variance of the true effect sizes and presents an estimate of the between-study variance in a random-effects model (*Cochrane handbook for systematic reviews of interventions,* 2020). Ultimately, we used strategies developed to address heterogeneity, such as rechecking the data and conducting subgroup analyses (Deeks JJ, 2019).

Additionally, when several autonomous study groups were compared with a single control group, (Chehrehnegar et al., 2019; Crawford et al., 2019; Heuer et al., 2013; Holden et al., 2018; Peltsch et al., 2014; Wilcockson et al., 2019; Yang et al., 2011, 2013) or when the effects were calculated over various time periods in the same sample (Crawford et al., 2015), the calculation of the average effect size that decreases over the observations would result in the omission of essential moderator information and would therefore not be appropriate. Accordingly, effect sizes for each of these nonindependent comparisons were included. To avoid underestimating the error variance associated with each effect size, the sample sizes used to calculate the standard errors for each group were divided by the number of their inclusions (*Cochrane handbook for systematic reviews of interventions,* 2020).

### Risk of Bias Across Studies

Publication bias was estimated by visual inspection of a funnel plot and Egger’s linear regression test (significant at *P* < 0.1) (Egger et al., 1997). Statistical analyses were conducted using Review Manager Version 5.3 software (Cochrane, London, UK: The Cochrane Collaboration, 2014) and JASP Team (2020) JASP (Version 0.13.1) [Computer software].

### Additional Analyses

Subgroup analyses were conducted to determine whether paradigm, clinical diagnosis (MCI and ADD), and outcomes (latency and error rate) in saccade paradigms contributed to the observed effect sizes. Chi^2^, I^2^, and Tau^2^ values were calculated to detect and quantify the heterogeneity across studies. All statistical analyses were performed using Review Manager software, version 5.3 (RevMan 5.3) and JASP computer software, version 0.13.1. Whenever a meta-analysis was not feasible because of a limited number of studies, a narrative summary was produced.

## Results

### Study Selection

The database search generated 5887 references of which 738 were duplicates, resulting in a total of 5149 unique articles. A total of 4966 were excluded because these studies did not meet the selection criteria. Subsequently, 183 full texts were assessed for eligibility, and 148 studies were excluded after full-text review based on our inclusion criteria. Subsequently, 36 studies met the eligibility criteria; however, two studies from the same research group had identical numerical outcomes (Crawford et al., 2005, 2013); therefore, only the later study (Crawford et al., 2013) was included in the final 35 studies included in the synthesis. Of these, eight studies (Bourgin et al., 2018; Bylsma et al., 1995; Currie et al., 1991; Mosimann et al., 2004; Pavisic et al., 2017; L. F. Scinto et al., 1994; T. Shakespeare et al., 2015; Verheij et al., 2012) did not meet the data availability inclusion criteria as the reported saccade paradigm temporal format could not be distinguished or gap and the overlap results were combined; thus, these studies were excluded from the meta-analysis. Thus, the remaining 27 studies (Abel et al., 2002; Alichniewicz et al., 2013; Boucart et al., 2014a, b; Boxer et al., 2006, 2012; Chehrehnegar et al., 2019; Crawford et al., 2013, 2015, 2019; de Boer et al., 2016; Garbutt et al., 2008; Hershey et al., 1983, 2013; Holden et al., 2018; Kaufman et al., 2012; Laurens et al., 2019; Lenoble et al., 2015, 2018; Mosimann et al., 2005; Noiret et al., 2018; Peltsch et al., 2014, 2020; Shafiq-Antonacci et al., 2003; Wilcockson et al., 2019; Yang et al., 2011, 2013) were included in the quantitative analysis (meta-analysis: see Fig. 1). Of the 34 studies included in the qualitative synthesis, 31 had defined saccade conditions, with twenty-four (77%) conducting ET in the gap condition.

### Study Characteristics

Of the 35 studies included in this review, 8 (23%) (Boucart et al., 2014a, b; Bourgin et al., 2018; Holden et al., 2018; Laurens et al., 2019; Lenoble et al., 2015, 2018; Noiret et al., 2018) were conducted in France, 7 (20%) (Boxer et al., 2006, 2012; Bylsma, 1995; Garbutt et al., 2008; Hershey et al., 1983; Heuer et al., 2013; L. F. M. Scinto et al., 1994) in the United States, 8 (23%) in the United Kingdom (Crawford et al., 2013, 2015, 2019; Mosimann et al., 2005; Pavisic et al., 2017; Polden et al., 2020; T. Shakespeare et al., 2015; Wilcockson et al., 2019), and the rest (34%) in Australia (Abel et al., 2002; Currie et al., 1991; Shafiq-Antonacci et al., 2003), Germany (Alichniewicz et al., 2013), Canada (Kaufman et al., 2012; Peltsch et al., 2014), China (Yang et al., 2011, 2013), the Netherlands (de Boer et al., 2016; Verheij et al., 2012) Switzerland (Mosimann et al., 2004) and Iran (Chehrehnegar et al., 2019). Two studies (Bylsma, 1995; Crawford et al., 2015) were longitudinal prospective cohort studies, whereas the rest were matched case–control studies.

The total sample size of the 35 included studies comprised 2435 subjects, 1252 controls and 1183 patients (386 MCI and 797 ADD patients). All the studies that reported on gender had both male and female participants. The characteristics of the included studies are described in Table 1.

### Risk of Bias Within Studies

Of the 35 studies assessed using the ROBINS-I risk of bias assessment tool (Table S4 in the Online Resource), 25 studies were rated as a moderate risk of bias (Abel et al., 2002; Alichniewicz et al., 2013; Boucart et al., 2014b; Bourgin et al., 2018; Boxer et al., 2006; Bylsma, 1995; Chehrehnegar et al., 2019; Crawford et al., 2015; Crawford et al., 2019; Currie et al., 1991; de Boer et al., 2016; Hershey et al., 1983; Holden et al., 2018; Kaufman et al., 2012; Laurens et al., 2019; Lenoble et al., 2018; Mosimann et al., 2004; Mosimann et al., 2005; Peltsch et al., 2014; L. F. M. Scinto et al., 1994; Shafiq-Antonacci et al., 2003; T. J. Shakespeare et al., 2015; Verheij et al., 2012; Wilcockson et al., 2019; Yang et al., 2011). Ten studies were rated as having a low risk of bias (Boucart et al., 2014a; Boxer et al., 2012; Crawford et al., 2013; Garbutt et al., 2008; Heuer et al., 2013; Lenoble et al., 2015; Noiret et al., 2018; Pavisic et al., 2017; Polden et al., 2020; Yang et al., 2013).

### Synthesis of Results

1. Qualitative Synthesis

We performed a qualitative analysis using the variables that were reported in most of the included studies. The common parameters for analysis were latencies and gain or amplitude in prosaccade and antisaccade and error rate in the antisaccade paradigm. The variables were analyzed according to differences observed between patients and controls. The analysis focused on the parameters excluded from the meta-analysis and the most widely reported parameters, in order to prevent repetition of the synthesis. A summary of analyzed articles is listed in Table 1.

1.1 Latency

Most studies placed the target stimuli in the horizontal plane. Of these, 14 studies also reported placing the target stimuli in the vertical plane separately or in combination with the horizontal plane target stimuli.

In twenty-four studies the saccade latency of patient groups (MCI and ADD) was compared with that of controls using gap conditions (Abel et al., 2002; Boucart et al., 2014a; Boucart et al., 2014b; Boxer et al., 2006; Boxer et al., 2012; Chehrehnegar et al., 2019; Crawford et al., 2015; Crawford et al., 2013; Crawford et al., 2019; de Boer et al., 2016; Garbutt et al., 2008; Heuer et al., 2013; Holden et al., 2018; Lenoble et al., 2015; Lenoble et al., 2018; Mosimann et al., 2004; Mosimann et al., 2005; Pavisic et al., 2017; Peltsch et al., 2014; Polden et al., 2020; T. J. Shakespeare et al., 2015; Wilcockson et al., 2019; Yang et al., 2011; Yang et al., 2013). Fourteen studies used overlap conditions (Boxer et al., 2006; Boxer et al., 2012; Chehrehnegar et al., 2019; Crawford et al., 2015; Crawford et al., 2013; Garbutt et al., 2008; Laurens et al., 2019; Mosimann et al., 2004; Mosimann et al., 2005; Peltsch et al., 2014; Polden et al., 2020; T. Shakespeare et al., 2015; Yang et al., 2011; Yang et al., 2013). Eight studies used step conditions (Abel et al., 2002; Alichniewicz et al., 2013; Currie et al., 1991; Hershey et al., 1983; Holden et al., 2018; Kaufman et al., 2012; Noiret et al., 2018; Shafiq-Antonacci et al., 2003). In 4 studies the variation could not be determined (Bourgin et al., 2018; Bylsma et al., 1995; L. F. Scinto et al., 1994; Verheij et al., 2012).

1.1.1 Prosaccade Latency

Thirty studies reported the prosaccade latency of controls compared to patients (Abel et al., 2002; Alichniewicz et al., 2013; Boucart et al., 2014a; Boucart et al., 2014b; Bourgin et al., 2018; Boxer et al., 2006; Boxer et al., 2012; Bylsma, 1995; Chehrehnegar et al., 2019; Crawford et al., 2015; Crawford et al., 2013; de Boer et al., 2016; Garbutt et al., 2008; Hershey et al., 1983; Heuer et al., 2013; Holden et al., 2018; Laurens et al., 2019; Lenoble et al., 2015; Lenoble et al., 2018; Mosimann et al., 2004; Mosimann et al., 2005; Noiret et al., 2018; Peltsch et al., 2014; Polden et al., 2020; L. F. M. Scinto et al., 1994; Shafiq-Antonacci et al., 2003; T. J. Shakespeare et al., 2015; Verheij et al., 2012; Yang et al., 2011; Yang et al., 2013) Nine of these studies had an MCI group alone (Alichniewicz et al., 2013) or with an ADD group (Chehrehnegar et al., 2019; Heuer et al., 2013; Holden et al., 2018; Laurens et al., 2019; Peltsch et al., 2014; Polden et al., 2020; Yang et al., 2011; Yang et al., 2013). Overall, 87% of the studies found that patients had a longer latency than controls, with no study reporting significantly longer latency in the control group.

1.1.2 Antisaccade Latency

There were 15 studies (Alichniewicz et al., 2013; Bourgin et al., 2018; Boxer et al., 2006; Boxer et al., 2012; Chehrehnegar et al., 2019; Crawford et al., 2013; Crawford et al., 2019; Currie et al., 1991; Garbutt et al., 2008; Heuer et al., 2013; Holden et al., 2018; Mosimann et al., 2005; Noiret et al., 2018; Peltsch et al., 2014; Shafiq-Antonacci et al., 2003; Wilcockson et al., 2019) that reported antisaccade latency patients compared with the controls. Eight of these studies had an MCI group alone (Alichniewicz et al., 2013) or with an ADD group (Chehrehnegar et al., 2019; Crawford et al., 2019; Heuer et al., 2013; Holden et al., 2018; Laurens et al., 2019; Peltsch et al., 2014; Wilcockson et al., 2019). Overall, 80% of the studies found that patients had a longer latency than controls, with no study reporting significantly longer latency in the control group.

1.1.3 Antisaccade Error Latency

Of the 7 studies (Bourgin et al., 2018; Boxer et al., 2006; Crawford et al., 2013; Crawford et al., 2019; Garbutt et al., 2008; Heuer et al., 2013; Noiret et al., 2018) that reported on the latency of error responses (prosaccades during antisaccade tasks), only one (Heuer et al., 2013) had an MCI group. Overall, 100% of studies found that patients had a longer latency than controls.

1.2 Antisaccade Error Rate

Of the 14 studies (Alichniewicz et al., 2013; Bourgin et al., 2018; Boxer et al., 2006; Boxer et al., 2012; Crawford et al., 2013; Garbutt et al., 2008; Heuer et al., 2013; Holden et al., 2018; Kaufman et al., 2012; Mosimann et al., 2005; Noiret et al., 2018; Peltsch et al., 2014; Shafiq-Antonacci et al., 2003; Wilcockson et al., 2019) that reported on the antisaccade error rate or correct antisaccades, only 5 studies (Alichniewicz et al., 2013; Holden et al., 2018; Peltsch et al., 2014; Wilcockson et al., 2019) had an MCI comparison group. Overall, 100% of the studies found that patients had a higher frequency of antisaccade errors than controls.

1.3 Gain or Amplitude

We examined gain or amplitude in both PS and AS. Overall, 10 (91%) studies (Boxer et al., 2012; Bylsma, 1995; Chehrehnegar et al., 2019; Crawford et al., 2015; Crawford et al., 2013; Garbutt et al., 2008; Mosimann et al., 2004; Mosimann et al., 2005; L. F. M. Scinto et al., 1994; Shafiq-Antonacci et al., 2003) studies reporting on gain or amplitude found hypometric saccades in patients. In 9 of these studies, comparisons were made only between age-matched controls and patients with ADD. Only one study (Chehrehnegar et al., 2019) with both MCI and ADD patient groups compared their reported findings to similar findings in other studies (hypometric saccades in patients). Overall, 90% of the studies found that compared to controls, patients had hypometric saccades, with no study reporting significantly smaller amplitudes in the control group.

2. Meta-analysis (Quantitative Analysis)

We conducted a meta-analysis derived from the visually guided and antisaccade paradigms of each saccade condition, comparing saccades in patient groups (combining MCI and ADD) and healthy age-matched controls. In order to compare studies with ADD patient groups to studies with MCI groups, outcomes (latency and error rate), and paradigms (prosaccade and antisaccade), we conducted subgroup-analyses. The effect sizes were calculated (from the study mean and standard deviation) as standardized mean differences and expressed as Hedges’ g (unbiased) using a random-effects model.

2.1 Gap

The first stage of the meta-analysis included 54 effect sizes of the gap condition that were derived from latency measures in the visually guided paradigms and latency and frequency of errors in the antisaccade paradigm for controls and patients (MCI and ADD) groups together. The overall weighted mean effect size in the gap condition was moderate (SMD: 0.52, CI: [0.37, 0. 68], Chi^2^ = 210.12, df = 53, *p* < 0.001, Tau^2^ = 0.24, I^2^ = 75 %) (Fig. A1 in the Online Resource). The I^2^ values indicated substantial heterogeneity; therefore, the presence of potential moderators.

Accordingly, in the second stage of analysis, we used the paradigm type (prosaccade and antisaccade) as a moderator variable. The subgroup analysis revealed the following (prosaccade, k = 27, Chi^2^ = 51.10, df = 26, p = 0.002, Tau^2^ = 0.09, I^2^ = 49%; antisaccade, k = 27, Chi^2^ = 141.70, df = 26, *p* < 0.001, Tau^2^ = 0.31, I^2^ = 82%).

**Fig. 4:**
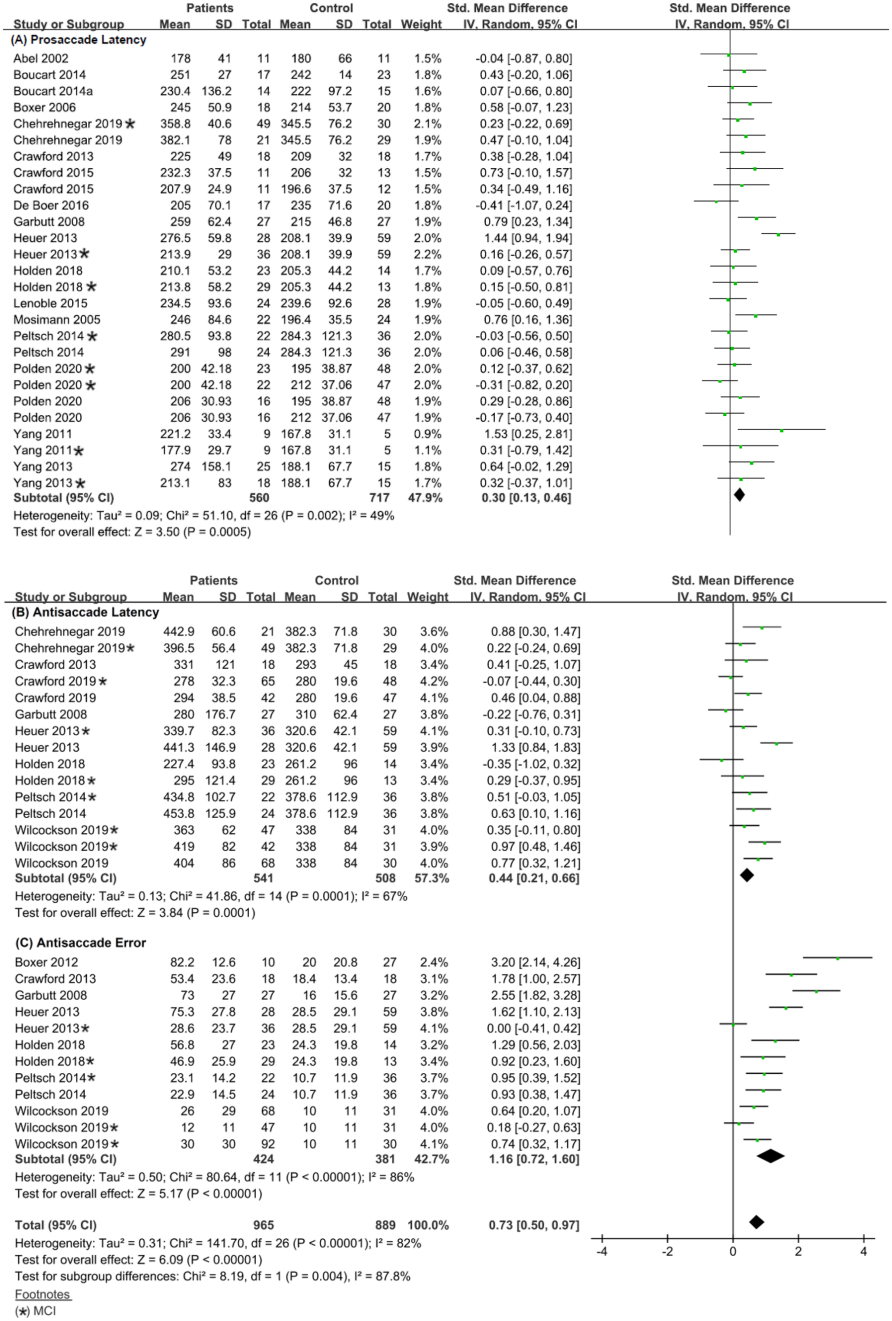
Forest plot of effect sizes and their confidence intervals comparing patients and controls in the gap condition for (**A**) prosaccade latency (msec), (**B**) antisaccade latency (msec), and (**C**) antisaccade error rate (%)

For the prosaccade group, the I^2^ value indicated low heterogeneity; therefore, the mean effect size was considered the best estimation for the data. In prosaccade studies, the overall weighted mean effect size in all studies was moderate (SMD: 0.30, CI: [0.13, 0.46] and MD: 15.88, CI: [7.42, 24.34]), suggesting a significant difference in prosaccade latency between the patient and control groups (Fig4A).

Subgroup analysis of prosaccade paradigm using the clinical diagnosis (MCI and ADD) as a moderator revealed the following: ADD group, k = 19, Chi^2^ = 47.6, df = 16, *p* < 0.001, Tau^2^ = 0.19, I^2^ = 66%; MCI group, k = 8, Chi^2^ = 1.14, df = 5, p = 0.95, Tau^2^ = 0.000, I^2^ = 0%. The I^2^ value indicated moderate heterogeneity in the ADD group and homogeneity for the MCI group; therefore, the mean effect size was considered the best estimation for the data. The overall weighted mean effect size in ADD studies was moderate (SMD: 0.39, CI: [0.17, 0.62] and MD: 21.37, CI: [9.80, 32.93]), and in MCI studies was small (SMD: 0.09, CI: [0.10, 0.28] and MD: 3.98, CI: [-4.58, 12.55]). This suggests that patients with ADD had significantly longer saccadic latencies when compared to controls whereas there were no significant differences between patients with MCI and controls (Fig. A2a, b in the Online Resource). Comparing prosaccade latency directly between patients with MCI and patients with ADD, revealed the following: k = 8, Chi^2^ = 19.66, df = 7, p = 0.006, Tau^2^ = 0.18, I^2^ = 64%. The I^2^ value indicated moderate heterogeneity; therefore, the mean effect size was considered the best estimation for the data. The overall weighted mean effect size between ADD and MCI was moderate (SMD: 0.45, CI: [0.08, 0.81] and MD: 24.03, CI: [4.78, 43.27]), suggesting a significant difference in saccadic reaction times between patients with ADD and patients with MCI in the prosaccade paradigm (Fig. A2c) in the Online Resource).

For the antisaccade group, the Chi^2^ and I^2^ values indicated the presence of substantial heterogeneity; therefore, the presence of potential moderators. In the antisaccade studies, the mean overall effect size was moderate (SMD: 0.73, CI: [0.50, 0.97]).

Subgroup analysis of the antisaccade paradigm using the outcomes (latency and error rate) as a moderator revealed the following (for latency, k = 15, Chi^2^ = 41.86, df = 14, *p* < 0.001, Tau^2^ = 0.13, I^2^ = 67%; for error rate, k = 12, Chi^2^ = 80.64, df = 11, *p* < 0.001, Tau^2^ = 0.50, I^2^ = 86%). For the latency outcome, the I^2^ value indicated moderate heterogeneity, therefore, the mean effect size was thus regarded as the best estimate for the data. In the error studies, the I^2^ values indicated substantial heterogeneity; therefore, the presence of potential moderators. In the antisaccade studies, the mean overall effect size in latency studies was moderate (SMD: 0.44, CI: [0.21, 0.66] and MD: 34.37, CI: [16.94, 51.80]), (Fig. 4B) whereas the mean overall effect size in error rate studies was large (SMD: 1.16, CI: [0.72, 1.60] and MD: 26.10, CI: [15.35, 36.84]), (Fig. 4C). This suggests a significant difference in outcome measures of saccade latency and frequency of errors between patients and controls.

Subgroup analysis of the antisaccade latency outcome using clinical diagnosis as the moderator revealed the following: ADD group: k = 8, Chi^2^ = 42.23, df = 7, *p* < 0.001, Tau^2^ = 0.28, I^2^ = 83%; MCI group: k = 7, Chi^2^ = 15.16, df = 6, p = 0.02, Tau^2^ = 0.07, I^2^ = 60%. In the ADD studies, the I^2^ values indicated substantial heterogeneity; therefore, the presence of potential moderators. The I^2^ value indicated moderate heterogeneity for the MCI group; therefore, the mean effect size was considered the best estimation for the MCI latency data (Fig. A3a, b in the Online Resource). In the ADD studies, the mean overall effect size in latency studies was moderate (SMD:0.55, CI: [0.15,0.95] and MD:40.47, CI: [10.19,70.75]), and the mean overall effect size in MCI studies was moderate (SMD:0.35, CI: [0.10, 0.60 and MD:28.55, CI: [6.14, 50.96]), suggesting that both patients with ADD patients with MCI had significantly different antisaccade saccade latency from healthy controls. In the additional analysis of the antisaccade paradigm comparing between patient groups (MCI vs. ADD), antisaccade latency revealed the following: k = 7, Chi^2^ = 24.37, df = 6, *p* < 0.001, Tau^2^ = 0.19, I^2^ = 75%. The I^2^ value indicated high heterogeneity; therefore, the presence of potential moderators. Between MCI and ADD, the overall weighted mean effect size was moderate (SMD: 0. 30, CI: [-0.07, 0.67] and MD: 20.70, CI: [-6.44, 47.85]), suggesting no significant differences in antisaccade latency between patients with ADD and patients with MCI. (Fig. A3c in the Online Resource).

Subgroup analysis of the error rate outcome using clinical diagnosis as the moderator revealed the following, the following was found: ADD group: k = 7, Chi^2^ = 33.97, df = 6, *p* < 0.001, Tau^2^ = 0.36, I^2^ = 82%; MCI group: k = 5, Chi^2^ = 15.57, df = 4, p = 0.004, Tau^2^ = 0.17, I^2^ = 74%. In the ADD group, the I^2^ value indicated high heterogeneity; therefore, the presence of potential moderators. In the MCI group, the I^2^ indicated moderate heterogeneity and consequently was considered the best estimate for data (Fig. A4a, b in the Online Resource). In the ADD studies, the mean overall effect size in error studies was large (SMD: 1.59, CI: [1.09, 2.09] and MD: 36.46, CI: [22.05, 50.86]), and the mean overall effect size in MCI studies was moderate (SMD: 0.55, CI: [0.14, 0.97] and MD: 10.98, CI: [2.58, 19.38]), suggesting that both patients with ADD patients with MCI had significantly higher frequency of errors compared to healthy controls. In the analysis of the error rate outcome between MCI vs. ADD, antisaccade error rate revealed the following: k = 5, Chi^2^ = 32.15, df = 4, *p* < 0.001, Tau^2^ = 0.46, I^2^ = 88%. The I^2^ value indicated high effect size heterogeneity and the presence of additional moderator(s); the overall weighted mean effect size was moderate (SMD: 0.53, CI: [-0.11, 1.17] and MD: 13.02, CI: [-3.36, 29.40]), (Fig. A4c in the Online Resource).

2.2 Step

The first stage of the meta-analysis included 12 effect sizes of the step condition that were derived from the visually guided and antisaccade paradigms for MCI and ADD groups together (Chi^2^ = 14.54, df = 11, p = 0.20, Tau^2^ = 0.05, I^2^ = 24%). The Chi^2^ and I^2^ values indicated homogeneity; therefore, the mean effect size was considered the best estimation for the data. The overall weighted mean effect size was large (SMD: 0.84, CI: [0.59, 1.08]), suggesting significant differences in outcomes between patients and healthy age matched controls (Fig. B1 in the Online Resource).

**Fig. 5:**
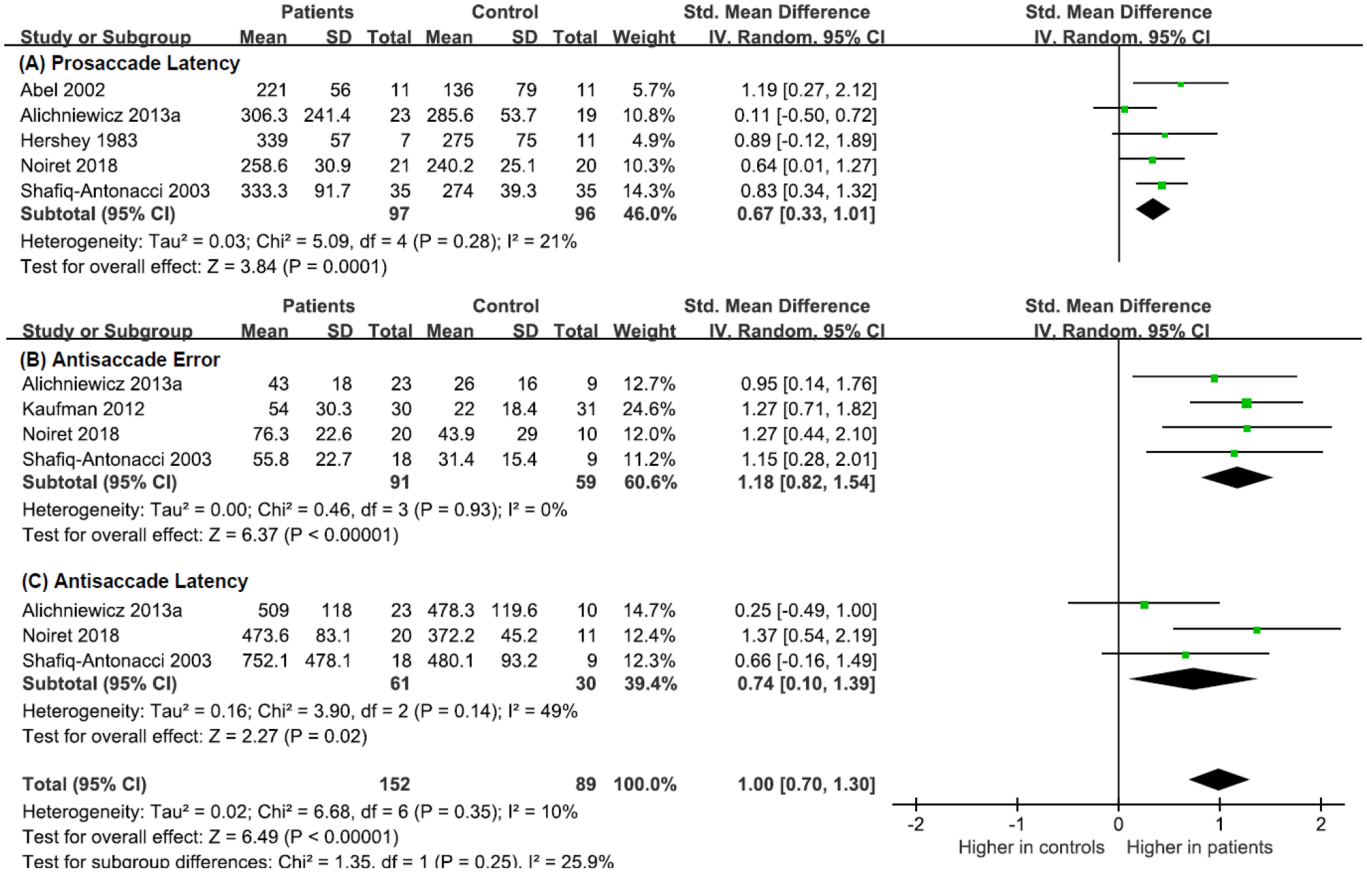
Forest plot of effect sizes and their confidence intervals comparing patients and controls in the step condition for (**A**) prosaccade latency (msec), (**B**) antisaccade error rate (%), and (**C**) antisaccade latency (msec)

Accordingly, in the second stage of analysis, we used the paradigm type as a subgroup moderating variable (prosaccade, k = 5, Chi^2^ = 5.09, df = 4, p = 0.28, Tau^2^ = 0.03, I^2^ = 21% (Fig. 5A); antisaccade, k = 7, Chi^2^ = 6.68, df = 6, p = 0.35, Tau^2^ = 0.02, I^2^ = 10%). The Chi^2^ value indicated homogeneity, and the I^2^ value indicated homogeneity; therefore, the mean effect size was considered the best estimation for the data. In prosaccade studies, the overall weighted mean effect size in MCI and ADD studies was moderate (SMD: 0.67, CI: [0.33, 1.01] and MD: 46.98, CI: [17.30, 76.66]), (Fig. 5A), suggesting a significant difference in saccadic latency between patients and controls. In the overall antisaccade studies, the mean overall effect size was large (SMD: 1.00, CI: [0.70, 1.30]), implying significant differences in outcome measures of latency and error rate between patients and controls. Due to the small number of studies, we did not perform subgroup analyses to compare healthy controls and patient groups separately.

In the subgroup analysis of the antisaccade, we used outcomes (latency and error rate) as a moderator (for error rate, k = 4, Chi^2^ = 0.45, df = 3, *p* = 0.93, Tau^2^ = 0.00, I^2^ = 0%, for latency, k = 3, Chi^2^ = 3.90, df = 2, *p* < 0.14, Tau^2^ = 0.16, I^2^ = 49%). The Chi^2^ value indicated homogeneity, and the I^2^ value indicated homogeneity and moderate homogeneity; thus, the mean effect size was considered the best approximation for the data. In studies with error rate as an outcome, the mean overall effect size was large (SMD: 1.18 CI: [0.82, 1.54] and MD: 25.52, CI: [18.13, 32.92]), suggesting a significant difference in the frequency of errors between patients and controls. In studies with latency as an outcome, the mean overall effect size was moderate (SMD: 0.74, CI: [0.10, 1.39] and MD: 93.55, CI: [12.75, 174.35), suggesting a significant difference in the saccadic reaction times between patients and controls (Fig. 5B).

2.3 Overlap

The first stage of the meta-analysis included 30 effect sizes of the overlap condition that were derived from the visually guided and antisaccade paradigms for MCI and ADD groups together (Chi^2^ = 83.67, df = 29, *p* < 0.001, Tau^2^ = 0.18, I^2^ = 65%). The I^2^ values indicated moderate heterogeneity; therefore, the mean effect size was considered the best estimation for the data. The overall weighted mean effect size was medium (SMD: 0.50, CI: [0.30, 0.69]), suggesting a significant difference between patients and controls (Fig. C1 in the Online Resource).

**Fig. 6:**
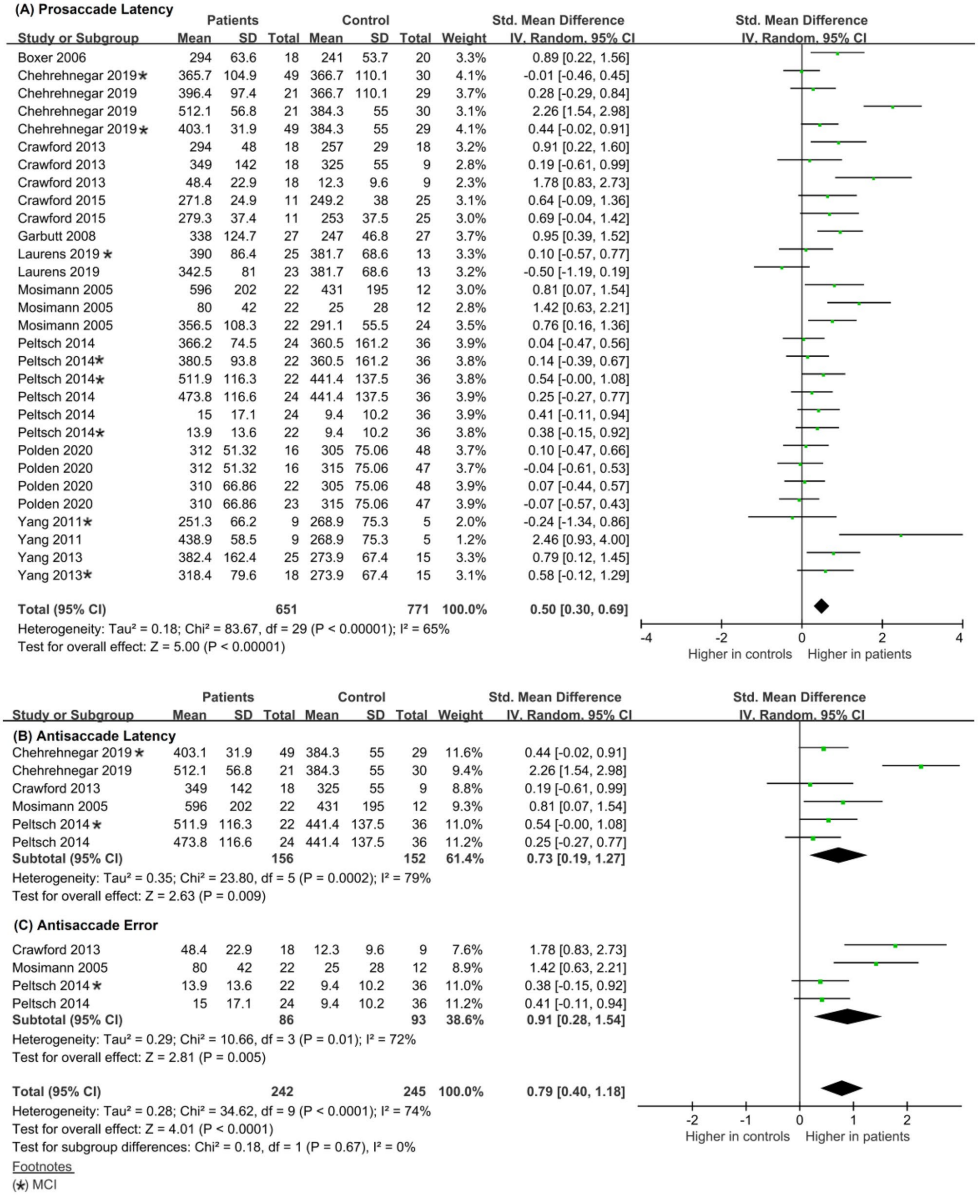
Forest plot of effect sizes and their confidence intervals comparing patients and controls in the overlap condition for (**A**) prosaccade latency (msec), (**B**) antisaccade latency (msec), and (**C**) antisaccade error (%)

Accordingly, in the second stage of analysis, we used the paradigm type as a subgroup moderator variable (prosaccade, k = 20, Chi^2^ = 39.79, df = 19, p = 0.003, Tau^2^ = 0.11, I^2^ = 52%; antisaccade, k = 10, Chi^2^ = 34.62, df = 9, *p* < 0.001, Tau^2^ = 0.28, I^2^ = 74%). For both groups, the I^2^ value indicated moderate heterogeneity; therefore, the mean effect size was considered the best estimation for the data. In the prosaccade overlap studies, the overall weighted mean effect size in MCI and ADD studies was moderate (SMD: 0.34, CI: [0. 14, 0.55]) and MD: 26.87, CI: [11.72, 42.01]), indicating that there was a significant difference in saccadic latency between the patient and control groups (Fig. 6A). In the antisaccade studies, the mean overall effect size was moderate (SMD: 0.79, CI: [0.40, 1.18]), suggesting a significant difference in outcomes (latency and error) between patients and controls.

Subgroup analysis of the prosaccade paradigm using clinical diagnosis (MCI and ADD) as a moderator revealed the following: ADD group, k = 13, Chi^2^ = 30.00, df = 12, p = 0.003, Tau^2^ = 0.16, I^2^ = 60%; MCI group, k = 7, Chi^2^ = 2.85, df = 6 p = 0.83, Tau^2^ = 0.00, I^2^ = 0%. The I^2^ value indicated moderate heterogeneity in the ADD group and homogeneity in the MCI group. In the ADD studies, the mean overall effect size was moderate (SMD: 0.50, CI: [0.22, 0.79] and MD: 36.78, CI: [16.53,57.03), whereas in MCI studies, it was small (0.08, CI: [-0.14,0.29] and MD: 6.88, CI: [10.69,24.45]), suggesting a significant difference in prosaccade latency between patients with ADD and controls, but no significant difference between patients with MCI and controls (Fig. C2a, b in the Online Resource). Additional analysis of the prosaccade comparing patient groups (MCI vs. ADD) using the same moderator revealed the following: k = 6, Chi^2^ = 22.64, df = 5, *p* < 0.001, Tau^2^ = 0.32, I^2^ = 78%. The I^2^ value indicated high effect size heterogeneity and the presence of additional moderator(s). Between the patient groups, the mean effect size was small (SMD: 0.26, CI: [-0.27, 0.79] and MD: 34.70, CI: [-23.25, 92.65]), suggesting no significant difference in the saccadic latency between patients with ADD and patients with MCI (Fig. C2c in the Online Resource).

For the antisaccade group, the Chi^2^ and I^2^ values indicated the presence of heterogeneity and high effect size heterogeneity and therefore the presence of additional moderator(s). In the analysis of the antisaccade paradigm, we used outcome (latency vs. error rate) as a subgroup moderating variable (for latency, k = 6, Chi^2^ = 24.32, df = 5, *p* < 0.001, Tau^2^ = 0.33, I^2^ = 79%, for error, k = 4, Chi^2^ = 17.34, df = 3, *p* < 0.001, Tau^2^ = 0.47, I^2^ = 83%)) (Fig. 6B). In the antisaccade studies, the mean overall effect size in latency studies was moderate (SMD: 0.73, CI: [0.19, 1.27] and MD: 66.05, CI: [12.65, 119.45]) whereas in error studies, the mean overall effect size was large (SMD: 0.91, CI: [0. 28, 1.54] and MD: 22.42, CI: [5.00,39.84]), suggesting a significant difference in frequency of errors between patients and healthy age matched controls (Fig. 6B).We did not perform further subgroup analyses due to the small number of studies.

### Gap Effect

#### Gap Effect for Controls

**Fig. 7:**
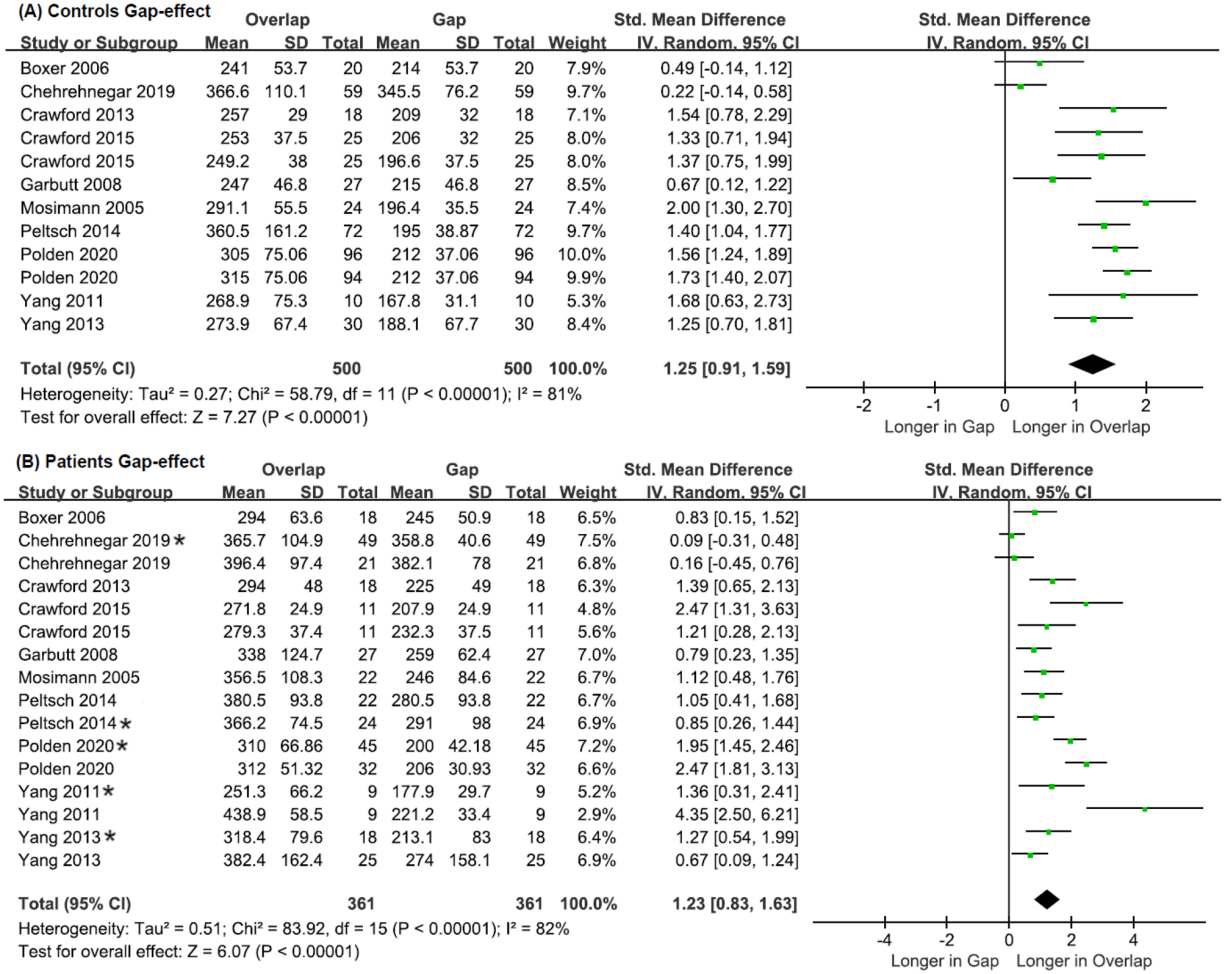
Forest plots of effect sizes and their confidence intervals, comparing prosaccade latency (msec) between overlap and gap conditions for (**A**) controls and (**B**) patients

The meta-analysis included 12 effect sizes that were derived from the visually guided and antisaccade paradigms for control groups together (Chi^2^ = 58.79, df = 11, *p* < 0.001, Tau^2^ = 0.27, I^2^ = 81%). The I^2^ values indicated substantial heterogeneity; therefore, the presence of additional moderators. In control studies, the overall weighted mean effect size was large (SMD: 1.25, CI: [0.91, 1.59] and MD: 85.80, CI: [51.24, 91.44]; Fig. 7A).

#### Gap Effect for Patients

The first stage of the meta-analysis included 16 effect sizes that were derived from the visually guided and antisaccade paradigms for patient groups together (Chi^2^ = 83.90, df = 15, *p* < 0.001, Tau^2^ = 0.51, I^2^ = 82%). The I^2^ values indicated substantial heterogeneity; therefore, the presence of additional moderators. In patient studies, the overall weighted mean effect size was large (SMD: 1.23, CI: [0.83, 1.63] and MD: 82.02, CI: [59.54, 105.50]; Fig. 7: B).

Subgroup analysis using clinical diagnosis as the moderator variable revealed the following: ADD, k = 11, Chi^2^ = 48.34, df = 10, *p* < 0.001, Tau^2^ = 0.49, I^2^ = 79%; MCI, k = 5, Chi^2^ =34.70, df = 4, *p* < 0.001, Tau^2^ = 0.71, I^2^ = 88%. For both patient groups, the I^2^ value indicated high heterogeneity; therefore, the presence of additional moderators. In both ADD and MCI patient studies, the overall weighted mean effect size was large: ADD (SMD: 1.29, CI: [0. 81, 1.76] and MD: 84.12, CI: [56.59, 111.64]), MCI (SMD: 1.12, CI: [0. 33, 1.92]) and MD: 77.9, CI: [31.61, 124.21]; Fig. D1a, b in the Online Resource).

### Anti-effect

#### Anti-effect for Controls

**Fig. 8:**
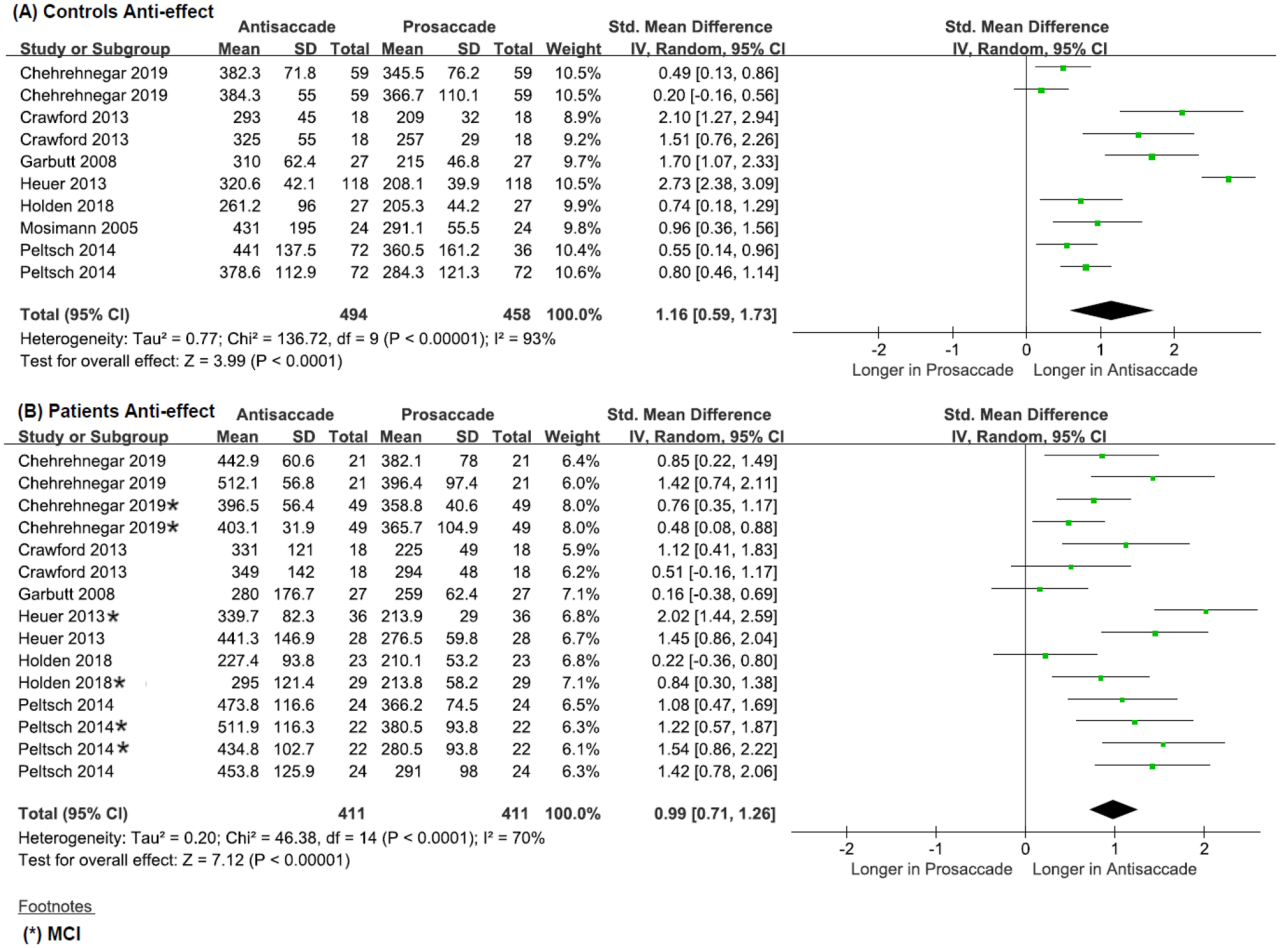
Forest plot of effect sizes and their confidence intervals, comparing latencies (msec) between antisaccade and prosaccade in gap and overlap conditions for (**A**) controls and (**B**) patients

The meta-analysis included 10 effect sizes that were derived from the visually guided and antisaccade paradigms for control groups together (Chi^2^ = 136.72, df = 9, *p* < 0.001, Tau^2^ = 0.77, I^2^ = 93%). The Chi^2^ and I^2^ values indicated high heterogeneity and therefore the presence of potential moderator(s). In control studies, the overall weighted mean effect size was large (SMD: 1. 16, CI: [0. 59, 1.73] and MD: 75.63, CI: [51.71, 99.55]) (Fig. 8A).

#### Anti-effect for Patients

The first stage of the meta-analysis included 15 effect sizes that were derived from the visually guided and antisaccade paradigms for patient groups together (Chi^2^ = 46.38, df = 14, *p* < 0.001, Tau^2^ = 0.20, I^2^ = 70%). The I^2^ values indicated moderate heterogeneity, therefore the mean effect size was regarded as the best approximation for the results. In patient studies, the overall weighted mean effect size was large (SMD: 0.99, CI: [0.71, 1.26] and MD: 89.86, CI: [63.66, 116.06]) (Fig. 8A), suggesting a significant difference in latency between antisaccade and prosaccade paradigms.

Subgroup analysis using clinical diagnosis as a moderator variable indicated the following: ADD, k = 9, Chi^2^ = 22.57, df = 8, p = 0.004, Tau^2^ = 0.19, I^2^ = 65%; MCI, k = 6, Chi^2^ = 23.05, df = 5, *p* < 0.001, Tau^2^ = 0.125, I^2^ = 78%. For the ADD group, the I^2^ value moderate heterogeneity; therefore, the mean effect size was regarded as the best approximation for the data. In both ADD patient studies and MCI patient studies, the overall weighted mean effect size was large, ADD: (SMD: 0.90, CI: [0.55, 1.25] and MD: 89.60, CI: [54.08, 125.13]), MCI :( SMD: 1.11, CI: [0.65, 1.57] and MD: 90.63, CI: [48.83, 132.43]) (Fig. E1a, b in the Online Resource). This suggests that there is a significant difference in the antisaccade and prosaccade latency when patient groups are compared independently.

### Summary

Overall, the results suggest that visually guided and antisaccade paradigms using gap, step and overlap conditions may be used to distinguish patients (MCI and ADD) from controls and MCI from ADD within patient groups when using prosaccade and antisaccade latency and error rate variables (Table 2). In addition, the magnitude of the effect size for both the gap effect and anti-effect is large in patients (MCI and ADD), similar to findings reported in healthy controls.

**Table 1:** Summary of studies that compared MCI or ADD patients to age-matched controls in saccade paradigms

Author, Year	Study Design	n	Sex (M/F)	Mean Age (yrs)	Diagnostic Criteria	Device (Brand, Company, Country)	Technique	Oculomotor Paradigm and Condition	Main Findings
Currie, J. 1991 (Currie et al., 1991)	Case–Control	C 15	8/7	43	NINCDS‐ADRDA (McKhann et al., 1984)	IRIS, Skalar, Delft, the Netherlands	Infrared; SRO	PS and AS	AS error rates correlated strongly with ADD severity.
		MCI 0	-	-					
		ADD 30	16/14	67 ± 8					
Crawford, T. J. 2015 (Crawford et al., 2015)	Prospective Cohort	C 25	8/17	70.6 ± 4.97	DSM-IV (APA, 2000), NINCDS‐ADRDA	“ExpressEye” (Optom, Freiburg, Germany)	Infrared; SRO	PS (Gap and Overlap, Go/No–Go)	Patients with ADD had a slower SRT than controls. After 12 months, both groups showed comparable reductions in latency to the gap stimulus compared to the overlap stimulus. Both groups showed general improvement, with more accurately directed saccades and faster reaction times over time.
		MCI 0	-	-					
		ADD 11	6/5	78.0 ± 4.94					
Lenoble, Q 2015 (Lenoble et al., 2015)	Case–Control	C 28	10/18	69.1 ± 7.1	NINCDS‐ADRDA (McKhann et al., 2011)	Red-M; SensoMotoric Instruments, Teltow, Germany	Infrared: PCR	PS (Gap)	Differences between YCs and OCs and patients with ADD were found in accuracy but not saccadic latency.
		MCI 0	-	-					
		ADD 24	10/14	71.4 ± 5.8					
Chehrehnegar, N. 2019 (Chehrehnegar et al., 2019)	Case–Control	C 59	23/36	62.55 ± 6.7	Petersen et al., 1999, DSM	SMI RED system (SensoMotoric Instruments)	Infrared: PCR	PS and AS (Gap and Overlap)	Saccadic gains were the most sensitive measures to distinguish MCI from controls. AS gap condition AUC = 0.7, PS gap condition AUC = 0.63, AS overlap condition AUC = 0.73. These parameters were strongly correlated with neuropsychological measures. Using tests in the parallel model, sensitivity was improved to 0.97.
		MCI 40	13/27	68.1 ± 8.8					
		ADD 31	16/15	67 ± 8					
Noiret, N. 2018 (Noiret et al., 2018)	Case–Control	C 20	9/11	71.75 ± 3.71	NINCDS‐ADRDA (McKhann et al., 2011)	ASL EYE-TRACK®6; Applied Science Laboratories; Bedford, MA	Infrared VOG	PS (Step, Predictive), AS (Step)	Patients with ADD had a higher SRT and SRT variability regardless of the task and had higher AS cost than the OC. Patients with ADD made more uncorrected ASs and took longer to correct incorrect ASs. In the PreS task, patients with ADD showed higher gain and gain variability than OC when they made anticipated saccades. Close relationships were found between the majority of SEM variables in PS, AS, and PreS tasks and dementia screening tests.
		MCI 0	-	-					
		ADD 20	9/11	79 ± 5.93					
Bourgin, J. 2018 (Bourgin et al., 2018)	Case–Control	C 25	7/18	71.24 ± 7.36	Croisile et al., 2012	Eyelink 1000 eye-tracker (SR Research, Kanata, ON, Canada)	Infrared: PCR	PS and AS	Controls made more AS errors for negative stimuli than for other stimuli and triggered PS toward negative stimuli more quickly than toward other stimuli. In contrast, patients with ADD showed no difference with regard to the emotional category in any of the tasks.
		MCI 0	-	-					
		ADD 16	7/9	74.14 ± 8.57					
Holden, J. G. 2018 (Holden et al., 2018)	Case–Control	C 27	13/14	69.5 ± 6.1	Dubois et al., 2004/07	EyeBRAIN® SURICOG	Infrared VOG	PS (Gap, Step) and AS (Gap)	Inhibitory impairments in stimulus-elicited saccades, characteristic of ADD, can be detected early in presumed prodromal patients using a simple, automated antisaccade task.
		MCI 29	11/18	71.3 ± 7.1					
		ADD 23	8/15	70.6 ± 6.1					
Lenoble, Q. 2018 (Lenoble et al., 2018)	Case–Control	C 12	6/6	70.2 ±6.8	NINCDS‐ADRDA (McKhann et al., 2011)	Red-M, SensoMotoric Instruments: Teltow Germany	Infrared: PCR	PS (Gap)	In contrast to both YC and OC, patients with ADD showed difficulties in refraining from a first saccade toward incongruent scenes in the free-viewing and implicit tasks.
		MCI 0	-	-					
		ADD 12	5/7	71.7 ± 5.9					
Pavisic, I. M. 2017 (Pavisic et al., 2017)	Case–Control	C 21	11/10	61.0 ± 5.3	NINCDS‐ADRDA (McKhann et al., 2011)	Eyelink II; SR Research	Infrared VOG	Fixation stability, PS, smooth pursuit	The ET paradigms of a relatively simple and specific nature provide measures not only reflecting basic oculomotor characteristics but also predicting higher-order visuospatial and visuoperceptual impairments.
		MCI 0	-	-					
		ADD 36	17/19	60.9 ± 5.2					
De Boer, C. 2016 (de Boer et al., 2016)	Case–Control	C 20	10/10	68.2 ± 6.3	NINCDS‐ADRDA (McKhann et al., 2011)	Chronos Vision, Berlin, Germany	Infrared VOG	PS	In two of three tasks, eye and hand timing and execution parameters significantly differed between groups. Such parameters could potentially give a quantitative description of disease-specific deficits in the spatial and temporal domains and may serve as a tool to monitor disease progression in ADD and PD populations.
		MCI 0	-	-					
		ADD 17	8/9	71.7 ± 3.5					
Shakespeare, T. J. 2015 (T. Shakespeare et al., 2015)	Case–Control	C 22	5/17	63.3 ± 6.2	Dubois et al., 2007/10	Eyelink II; SR Research	Infrared VOG	Fixation stability, PS (Gap, Overlap), sinusoidal pursuit	The greatest differences between PCA and typical ADD were seen in saccadic performance. Patients with PCA made significantly shorter saccades, especially for distant targets. They also exhibited a significant exacerbation of the normal gap/overlap effect, consistent with “sticky fixation”. The SRT was significantly associated with parietal and occipital cortical thickness measures.
		MCI 0	-	-					
		ADD 17	9/8	67.4 ±5.9					
Boucart, M. 2014 (Boucart et al., 2014a)	Case–Control	C 23	N/A	72 ± 7.5	NINCDS‐ADRDA (McKhann et al., 2011)	SensoMotoric Instruments: Teltow Germany)	Infrared VOG	PS (Gap)	Patients with ADD were significantly less accurate than age-matched controls, and older participants were less accurate than younger ones.
		MCI 0	-	-					
		ADD 17	N/A	70.2 ± 3.1					
Peltsch, A 2014 (Peltsch et al., 2014)	Case–Control	C 72	22/50	73 ± 6	Petersen et al., 1999, NINCDS‐ADRDA (McKhann et al., 1984)	Grass Technologies P18, General Purpose AC Amplifier, Warwick, RI, USA	Direct current EOG	PS and AS (Gap and Overlap)	In the AS task, the saccadic latency distributions of both the aMCI and ADD groups were markedly different from that of controls. The ADD group showed a distinct profile that was different from the aMCI and control curves in PS.
		aMCI 22	10/12	76 ± 8					
		ADD 24	9/15	76 ± 8					
Boucart, M 2014 (Boucart et al., 2014b)	Case–Control	C 15	5/10	66 ± 7	Dubois et al., 2010	RED-m, SensoMotoric Instruments, Berlin, Germany	Infrared: PCR	PS (Gap)	Participants with PCA were more impaired in detection of a target within a scene than participants with ADD.
		MCI 0	-	-					
		ADD 14	6/8	71.5 ± 10					
Yang, Q. 2013 (Yang et al., 2013)	Case–Control	C 30	15/15	73.8 ± 9.4	Petersen et al., 2001, Hachinski Ischemia Scale	Eyeseecam system (University of Munich Hospital, Clinical Neuroscience, Munich, Germany)	Infrared VOG	PS (Gap and Overlap)	There was a shorter SRT in gap than in overlap for the ADD, aMCI, and OC groups. The SRTs of saccades in gap and overlap conditions were abnormally long for ADD patients relative to OCs and aMCI patients. The SRT was longer for patients with aMCI than for OCs in the overlap condition. There was a significant correlation between MMSE scores and the SRT of saccades in the ADD group alone. Latency had a higher coefficient of variation for patients with ADD than for OCs or for patients with aMCI. Variability of accuracy and speed was abnormally high in patients with ADD—higher than that in patients with aMCI or in OCs. An abnormally long SRT of saccades in gap and overlap conditions was noted for ADD patients relative to OCs and patients with MCI.
		MCI 18	7/11	77.6 ± 10.7					
		ADD 25	7/18	73.5 ± 8.2					
Yang, Q. 2011 (Yang et al., 2011)	Case–Control	C 10	6/4	69.7 ± 6.1	Petersen et al., 1999, 2001/04, Dubois et al., 2007/10, DSM-IV (APA)	IRIS; Skalar, Delft, The Netherlands	Infrared VOG	Fixation and PS (Gap and Overlap)	The SRTs were shorter in the gap than in the overlap condition (a gap effect) in all three groups of subjects: OC, MCI and ADD. For both conditions, the SRT of saccades was longer for patients with ADD than for OC and MCI subjects. The accuracy and mean velocity were normal in MCI and ADD subjects; however, the variability in accuracy-speed was higher for patients with ADD than for OC and MCI subjects in the overlap condition.
		MCI 9	6/3	71.4 ± 9.8					
		ADD 9	4/5	68.7 ± 9.2					
Heuer, H. W. 2013 (Heuer et al., 2013)	Case–Control	C 118	49/69	69.4 ± 6.2	CDR, NINCDS‐ADRDA (McKhann et al., 1984)	Fourward Technologies, Gallatin, MO	Infrared: PCR DPI	PS and AS (Gap)	AS performance in patients with MCI resembled that in OCs. In all subjects, AS performance correlated with neuropsychological measures of executive function, even after controlling for disease severity. In subjects with MCI but not in OCs, cortical thickness in the frontoparietal AD signature regions was correlated with AS performance.
		MCI 36	18/18	72.9 ± 6.7					
		ADD 28	16/12	60.9 ± 8.7					
Alichniewicz, K. K. 2013 (Alichniewicz et al., 2013)	Case–Control	C 19	8/11	58.84 ± 7.41	Revised criteria for aMCI (Artero et al., 2006)	MR-Eyetracker (Cambridge Research Systems, Ltd)	Infrared: Limbus tracking	PS and AS (Step)	fMRI revealed decreased activation in the parietal lobe in OCs compared to YCs and decreased activation in the FEF in patients with MCI compared to OCs during the execution of ASs.
		MCI 23	5/18	60.30 ± 9.31					
		ADD 0	-	-					
Crawford, T. J. 2013 (Crawford et al., 2013)	Case–Control	C 18	8/10	75 ± 3.6	DSM-IV (APA 2000)	‘ExpressEye’ (Optom, Freiburg, Germany)	Infrared; SRO	PS and AS (Gap, Overlap)	Uncorrected errors in the AST were selectively increased in ADD but not in PD compared to the control groups. There was an increase in the SRT to targets that were presented simultaneously with the fixation stimulus compared to the removal of fixation. The gap effect was elevated in the OC compared to YC, showing a strong effect of aging and no specific effect of neurodegenerative disease.
		MCI 0	-	-					
		ADD 18	13/5	78 ± 4.8					
Boxer, A. L. 2012 (Boxer et al., 2012)	Case–Control	C 27	9/18	66.8 ± 8.3	NINCDS‐ADRDA (McKhann et al., 1984)	Fourward Technologies (Gallatin, MO) Generation 6.1	Infrared: PCR DPI	PS (Gap and Overlap) and AS (Gap)	All FTD and ADD subjects were impaired relative to OC on the AS task. However, only FTLD-tau and ADD patients displayed reflexive PS abnormalities. ADD patients displayed prominent increases in the horizontal saccade SRT that differentiated them from FTD cases. Impairments in velocity and gain were most severe in individuals with PSP but were also present in other tauopathies. Vertical and horizontal saccade velocity and gain were able to differentiate PSP patients from other patients. Vertical saccade velocity was strongly correlated with dorsal midbrain volume.
		MCI 0	-	-					
		ADD 10	9/1	60.4 ± 8.5					
Kaufman, L. D. 2012 (Kaufman et al., 2012)	Case–Control	C 31	13/18	70.5 ± 8.2	NINCDS‐ADRDA (McKhann et al., 1984)	Dell Inspiron 1520 Notebook 2.0 M pixel webcam	Infrared VOG	PS and AS (Step)	Patients with ADD made more AS errors and corrected fewer errors than age-matched controls. Error rates, corrected or uncorrected, were not correlated with the ADD MMSE or Dementia Rating Scale scores.
		MCI 0	-	-					
		ADD 30	19/11	72.3 ± 9.7					
Verheij, S. 2012 (Verheij et al., 2012)	Case–Control	C 18	9/9	69.8 ± 6.5	DSM-IV TR (APA, 2000), NINCDS‐ADRDA (McKhann et al., 1984)	Chronos Vision, Berlin, Germany	Infrared VOG	PS	Patients with ADD needed significantly more time than controls to initiate and execute goal-directed hand movements. Patients with ADD are also unable to suppress reflexive eye and, to a lesser extent, hand movements.
		MCI 0	-	-					
		ADD 16	6/10	75.4 ± 6.7					
Garbutt, S. 2008 (Garbutt et al., 2008)	Case–Control	C 27	10/17	65.0 ± 1.5	NINCDS‐ADRDA (McKhann et al., 1984)	Fourward Technologies (Buena Vista, VA, USA) Generation 6.1	Infrared: PCR DPI	PSs (Gap and Overlap), Smooth pursuit, AS (Gap)	Only PSP patients displayed abnormalities in saccade velocity, whereas abnormalities in saccade gain were observed in PSP>CBS>ADD subjects. All patient groups except those with SD were impaired on the AS task, but only FTLD subjects and not ADD, CBS or PSP subjects were able to spontaneously self-correct AS errors and controls.
		MCI 0	-	-					
		ADD 28	17/11	59.8 ± 1.4					
Boxer, A. L. 2006 (Boxer et al., 2006)	Case–Control	C 20	7/13	64.4 ± 7.2	NINCDS‐ADRDA (McKhann et al., 1984)	Generation 6.1; Fourward Systems, Roanoke, VA	Infrared: PCR DPI	Smooth Pursuit, PS (Gap and Overlap) and AS (Gap and Overlap)	Patients with clinical syndromes associated with dorsal frontal lobe damage had normal PS but were impaired relative to other patients and control subjects in smooth pursuit EMs and on the AS task. The percentage of correct AS responses was correlated with neuropsychological measures of frontal lobe function and with estimates of frontal lobe gray matter volume based on analyses of structural magnetic resonance images. An unbiased voxel-based morphometric analysis identified the volume of a segment of the right FEF as positively correlated with AS performance but not with either pursuit performance or AS or PS SRT or gain. In contrast, the volume of the pre-SMA and a portion of the SEF correlated with AS SRT but not with the percentage of correct responses.
		MCI 0	-	-					
		ADD 18	12/6	58.4 ± 7.2					
Mosimann, U. P. 2005 (Mosimann et al., 2005)	Case–Control	C 24	N/A	75.3 ± 5.8	NINCDS‐ADRDA (McKhann et al., 1984)	Eyelink™; SensoMotorik instruments, Berlin, Germany	Direct current EOG	PS (Gap, Overlap, Predictive, Decision), and AS	Patients with ADD were impaired only in complex saccade performance. Impaired saccade execution in reflexive tasks allowed discrimination between DLB and ADD and between PDD and Parkinson’s disease when ± 1.5 SD was used for group discrimination.
		MCI 0	-	-					
		ADD 22	N/A	78.1 ± 6.8					
Mosimann, U. P. 2004 (Mosimann et al., 2004)	Case–Control	C 24	15/9	72.9 ± 6.9	DSM-IV (APA, 1994), NINCDS‐ADRDA (McKhann et al., 1984)	Eyelink™; SensoMotorik Instruments, Berlin, Germany	Infrared VOG	PS (Gap and Overlap)	Patients with ADD had longer fixations and smaller saccade amplitudes than controls.
		MCI 0	-	-					
		ADD 24	11/13	74.3 ± 6.3					
Shafiq-Antonacci, R. 2003 (Shafiq-Antonacci et al., 2003)	Case–Control	C 35	79/166	62.8 ± 8.6	NINCDS‐ADRDA (McKhann et al., 1984)	IRIS; Skalar Medical BV, Delft, the Netherlands	Limbus infrared reflectance	PS (Random and Predictive) and AS	Patients had a longer and more variable SRT, more hypometric and anticipatory random saccades, and higher AS error rates than controls. The AS error rate was correlated with dementia severity. AS measures were the most specific, and random saccade gain was the most sensitive.
		MCI 0	-	-					
		ADD 35	15/20	70.9 ± 9.4					
Abel, L. A. 2002 (Abel et al., 2002)	Case–Control	C 11	6/5	67.36 ± 5.44	NINCDS‐ADRDA (McKhann et al., 1984)		Scleral coil and EOG	PS (Gap, Predictive), AS	As a group, patients’ SRTs were significantly higher and more variable than those of controls in the simultaneous and gap conditions. The mean PreS task performance was similar but significantly more variable. Grossly anticipatory responses by patients were common in the predictable, simultaneous and gap conditions. When making target-driven saccades, patients with ADD demonstrated a gap effect of similar magnitude to that of OC. Patients made significantly fewer correct ASs and significantly more reflexive errors not followed by a corrective AS than did controls.
		MCI 0	-	-					
		ADD 11	5/6	73.09 ± 9.39					
		MCI 0	-	-					
		ADD 10	N/A	68 ± 6.1					
Bylsma, F. W. 1995 (Bylsma et al., 1995)	Prospective Cohort	C: B-31, FU-17	16/15, 11/6	71.4 ± 5.6, 71.3 ± 6.4	NINCDS‐ADRDA (McKhann et al., 1984)	Sensor Medics Ag/AgCI 11-mm miniature electrodes	EOG	Fixation and PS (Predictive)	Patients with ADD and control subjects made equal numbers of intrusive saccades at baseline. A progressive increase in the number of intrusive saccades over 9- and 18-month intervals was noted only for patients with ADD, and this increase correlated with increased dementia severity, as indexed by MMSE scores. On the saccade task, the groups differed at baseline in the SRT after target displacement. The SRT showed no change over time and was not associated with increasing dementia severity.
		MCI 0	-	-					
		ADD 31, 17	16/15, 5/12	71.8 ± 6.1, 71.2 ± 6.3					
Scinto, L. F. 1994 (L. F. M. Scinto et al., 1994)	Case–Control	C: 11	2/9	71.1 ± 5.5	NINCDS‐ADRDA (McKhann et al., 1984)	Model 300, Applied Science Laboratories, Waltham, Mass.	Infrared: PCR	PS (Sequence, Voluntary)	Patients with probable ADD did not exhibit significantly different saccade latencies to the appearance of the target than normal controls.
		MCI 0	-	-					
		ADD 10	4/6	72.6 ± 4.3					
Hershey, L. A. 1983 (Hershey et al., 1983)	Case–Control	C: 11	N/A	72 ± 7.9	DSMIII	Beckman Type R rectilinear Dynograph.	Infrared VOG	PS	A prolonged SRT was present in patients with ADD and in those with other dementias. There appeared to be no correlation between SRT prolongation and cognitive impairment, as estimated by CCSE scores (r= .32) or MMSE scores (r = .17).
		MCI 0	-	-					
		ADD 7	1/6	72.3 ± 8.4					
Laurens, B., 2019 (Laurens et al., 2019)	Case–Control	C 26 aMCI 25 ADD 23	12/14 10/15 8/15	69.5 ± 6.1 71.3 ± 7.1 70.6 ± 6.1	Dubois et al., 2007, 2010	Eyebrain T1® (EBT1)	Infrared illumination	Volitional (Spatial Decision)	Patients with mild ADD made more errors on a spatial decision task than aMCI patients and controls. Impaired visuospatial judgment may explain these results and distinguish aMCI patients from mild ADD patients.
Wilcockson, T. D. W.2019 (Wilcockson et al., 2019)	Case–Control	C 92 naMCI 47 aMCI 42 ADD 68	40/52 27/20 17/25 34/34	69 ± 7.2 69 ± 6.9 74 ± 7.4 74 ± 7.7	NINCDS‐ADRDA) (McKhann et al., 1984)	EyeLink Desktop 1000 eye-tracker (SR Research)	Pupil-corneal reflection	AS	AST can discriminate between people with aMCI and naMCI; and replicated the previously reported impairment in inhibitory control of antisaccades in people with ADD.
Crawford, T. J.2019 (Crawford et al., 2019)	Case–Control	C 95 MCI 65 ADD 42	36/59 31/34 21/21	66.7 ± 8.6 70.5 ± 8.0 74.4 ± 7.8	NINCDS‐ADRDA) (McKhann et al., 1984)	EyeLink Desktop 1000 eye-tracker (SR Research)	Pupil-corneal reflection	AS	An overall increase in the frequency of AST errors in ADD and MCI compared to the control group was noted.
Polden, M.2020 (Polden et al., 2020)	Case–Control	C OEP 96 C SAP 94 MCI 45 ADD 32	-	66.18 ± 7.94 67.25 ± 6.13 70.83 ± 8.17 74.32 ± 7.57	DSM-IV (APA 2000), NINCDS‐ADRDA (McKhann et al., 2011)	EyeLink Desktop 1000 eye-tracker (SR Research)	Pupil-corneal reflection	PS (Gap and Overlap)	A reduction in the saccade latency was observed in all the participant groups in the gap condition compared to the overlap condition, confirming the gap effect. The gap effect was also preserved in participants with MCI and ADD.

*N/A* represents unavailable data, *P* patients, *C* controls, *ADD* Alzheimer’s disease dementia, *aMCI* amnestic mild cognitive impairment, *DLB* dementia with Lewy bodies, *naMCI* non-amnestic mild cognitive impairment, *MCI* mild cognitive impairment, *PCA* posterior cortical atrophy, *PD* Parkinson disease, *PSP* progressive supranuclear palsy, *FTLD* frontotemporal lobar degeneration, *YC* young control, *OC* old control, *PS* prosaccade, *AS* antisaccade, *AUC* area under the curve, *APA* American Psychiatric Association, *DSM* Diagnostic and Statistical Manual of Mental Disorders, *NINCDS-ADRDA* National Institute of Neurological and Communicative Disorders and Stroke–Alzheimer’s Disease and Related Disorders Association criteria for probable AD, *CCSE* Cognitive Capacity Screening Examination, *CDR* Clinical Dementia Rating, *MMSE* Mini-Mental State Examination, *EOG* electrooculography, *VOG* video-oculography, *DPI* dual Purkinje image, *PreS* predictive saccade, *SRT* saccadic reaction time, *SEM* saccadic eye movement, *pre-SMA* presupplementary motor area, *FEF* frontal eye field, *SEFs* supplementary eye fields, *fMRI* functional magnetic resonance imaging, *PCR* pupil-corneal reflection oculography, *SRO* scleral reflection oculography, *OEP* older European participants, *SAP* older South Asian participants

**Table 2:** Summary of meta-analysis results for saccadic eye movements

Condition	Saccade paradigm	Outcome	Comparison	k	Participants N_P_/N_C_	Effect estimates SMD (95% CI)	Z	*p* - value	Heterogeneity estimates				Reference Figure
									Chi^2^	*p* - value	I^2^ (%)	Tau^2^	
Gap	PS	Latency	C vs. P	27	560/717	0.30[0.13, 0.46]	3.50	< 0.001	51.10	0.002	49	0.09	Fig. 4A
			C vs. ADD	19	352/464	0.39[0.17, 0.62]	3.44	< 0.001	41.19	< 0.001	56	0.14	Fig. 2A
			C vs. MCI	8	208/253	0.09[-0.10, 0.28]	0.95	0.34	3.65	0.82	0	0.00	Fig. 2B
			MCI vs. ADD	8	208/162	0.45[0.08, 0.81]	2.40	0.02	19.66	0.006	64	0.18	Fig. 2C
	AS	Latency	C vs. P	15	541/508	0.44[0.21, 0.66]	3.84	< 0.001	41.86	< 0.001	67	0.13	Fig. 4B
			C vs. ADD	8	251/508	0.55[0.15, 0.95]	2.67	0.008	42.23	< 0.001	83	0.28	Fig. 3A
			C vs. MCI	7	290/463	0.35[0.10, 0.60]	2.79	0.005	15.16	0.02	60	0.07	Fig. 3B
			MCI vs. ADD	6	290/206	0. 30[-0.07, 0.67]	1.58	0.11	24.37	< 0.001	75	0.19	Fig. 3C
		Error	C vs. P	12	424/381	1.16[0.72, 1.60]	5.17	< 0.001	80.64	< 0.001	86	0.50	Fig. 4C
			C vs. ADD	7	198/381	1.59[1.09, 2.09]	6.18	< 0.001	33.97	< 0.001	82	0.36	Fig. 4A
			C vs. MCI	5	226/263	0.55[0.14, 0.97]	2.59	0.009	15.57	0.004	74	0.17	Fig. 4B
			MCI vs. ADD	5	143/226	0.53[-0.11, 1.17]	1.62	0.11	32.15	< 0.001	88	0.46	Fig. 4C
Step	PS	Latency	C vs. P	5	97/96	0.67[0.33, 1.01]	3.84	< 0.001	5.09	0.28	21	0.03	Fig. 5A
	AS	Latency	C vs. P	3	61/30	0.74[0.10, 1.39]	2.27	0.02	3.90	0.14	49	0.16	Fig. 5B
		Error	C vs. P	4	91/59	1.18[0.82, 1.54]	6.37	< 0.001	0.46	0.93	0	0.00	Fig. 5C
Overlap	PS	Latency	C vs. P	20	509/526	0.34[0.14, 0.55]	3.31	< 0.001	39.79	0.003	52	0.11	Fig. 6A
			C vs. ADD	13	241/332	0.50[0.22, 0.79]	3.44	< 0.001	30.00	0.003	60	0.16	Fig. 2A
			C vs. MCI	7	168/194	0.08[-0.14, 0.29]	0.70	0.48	2.85	0.83	0	0.00	Fig. 2B
			MCI vs. ADD	6	134/168	0.26[-0.27, 0.79]	0.98	0.33	22.64	< 0.001	78	0.38	Fig. 2C
	AS	Latency	C vs. P	6	156/173	0.72[0.21, 1.24]	2.75	0.006	24.32	< 0.001	79	0.33	Fig. 6B
		Error	C vs. P	4	86/114	1.04[0.29, 1.78]	2.73	0.006	17.34	< 0.001	83	0.47	Fig. 6C
Gap, Step, & Overlap	PS & AS	Gap-effect (gap vs. step/overlap)	C vs. C	12	500	1.25[0.91, 1.59]	7.29	< 0.001	58.79	< 0.001	81	0.27	Fig. 7A
			P vs. P	16	361	1.23[0.83, 1.63]	6.07	< 0.001	83.92	< 0.001	82	0.51	Fig. 7B
			ADD vs. ADD	11	218	1.29[0.81, 1.76]	5.26	< 0.001	48.34	< 0.001	79	0.49	Fig. 1A
			MCI vs. MCI	5	143	1.12[0.33, 1.92]	2.76	0.006	34.70	< 0.001	88	0.71	Fig. 1B
Gap, Step, & Overlap	PS & AS	Anti-effect (PS vs. AS)	C vs. C	10	494	1.16[0.59, 1.73]	3.99	< 0.001	136.7	< 0.001	93	0.77	Fig. 8A
			P vs. P	15	411	0.99[0.71, 1.26]	7.12	< 0.001	46.38	< 0.001	70	0.20	Fig. 8B
			ADD vs. ADD	9	204	0.90[0.55, 1.25]	5.03	< 0.001	22.75	0.004	65	0.19	Fig. 1A
			MCI vs. MCI	6	207	1.11[0.65, 1.57]	4.74	< 0.001	23.05	< 0.001	78	0.25	Fig. 1B

Some studies included patients with ADD and MCI, and each of these non-independent comparisons was included. The gap effect and anti-effect comparisons of participants reflect only within-group comparisons

*C* Controls, *ADD* Dementia due to Alzheimer’s disease, *MCI* Mild cognitive impairment,*SMD* Standardized mean difference, *k* Number of effect sizes, *P* Patients, *N*_*P* _Number of patients, N_C_ Number of controls

### Risk of Bias Across Studies

**Fig. 9:**
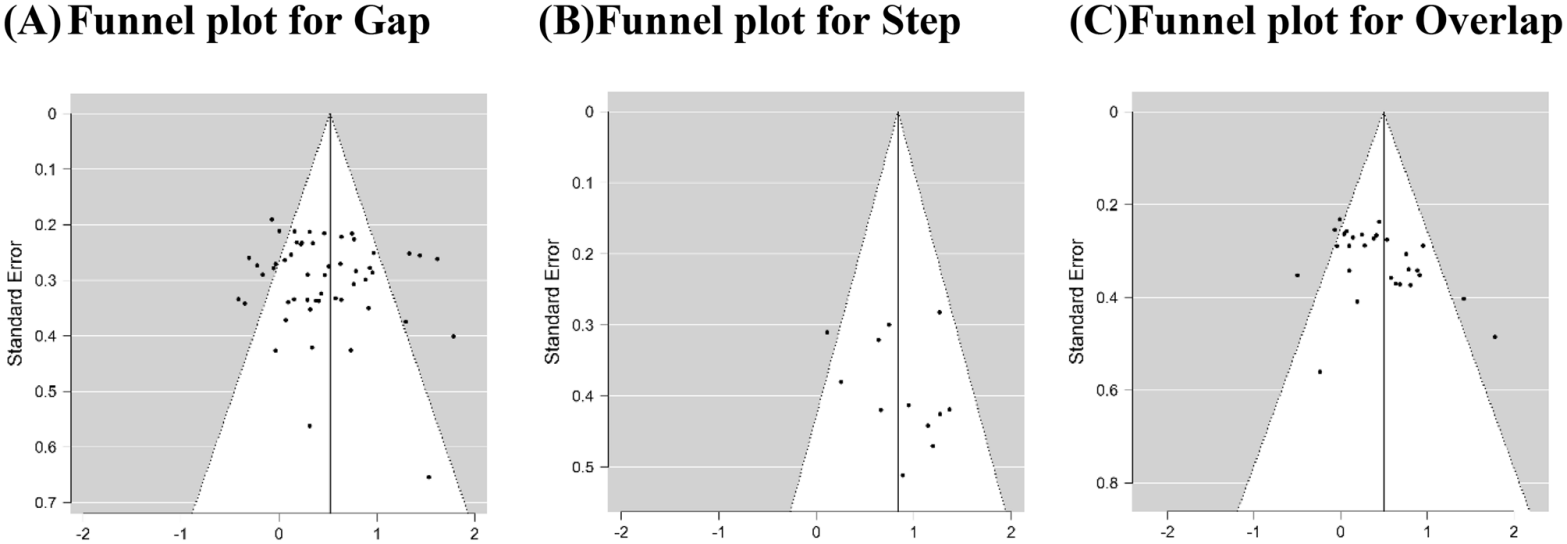
Funnel plot depicting the effect size (x axis) by their standard error (y axis) for (**A**) gap, (**B**) step, and (**C**) overlap

The funnel plot and Egger’s tests were conducted to evaluate the publication bias of this meta-analysis. The results indicated that publication bias for gap (Z = 2.603, *p* = 0.009) and overlap (Z = 3.368, *p* = 0.002) whereas no publication bias was identified, and the pooled results were stable (Z = 0.967, *p* = 0.334) for step (Fig. 9).

## Discussion

In this review, we assessed variations in saccadic EMs between patients (MCI and ADD) and healthy age-matched controls. We conducted a qualitative synthesis and a meta-analysis comparing saccadic performances based on (1) conditions (gap, step, and overlap) between interparticipant groups on the same paradigm, (2) gap effect (gap vs. step/overlap), in controls and patients (MCI and ADD) and (3) anti-effect (latencies in antisaccade vs. prosaccade), in controls and patient groups.

First, we examined saccades in controls and patients (MCI and ADD) together using (1) the gap condition, in which the fixation point is offset approximately 200 msec before the target comes on followed by (2) the step and (3) the overlap condition, in which the fixation point stays on after the target appears. The meta-analysis results showed that regardless of the condition (gap, step, or overlap), saccadic EMs may be used to distinguish control and patient groups (MCI and ADD). This may suggest that cognitive function is related to saccadic EM deficits, as both patient groups performed worse than controls.

Second, we used paradigms (prosaccade and antisaccade paradigms), outcomes (latency and error rate), and diagnosis (MCI and ADD) in the three conditions as moderators to ascertain if they contributed to the observed differences. Overall, in both saccadic paradigms, when we compared controls and patients, visually guided saccade paradigms revealed a moderate effect size, whereas the antisaccade paradigms indicated a large effect size. The larger effect size in antisaccade paradigms may reflect impaired processing and defective higher-order cognitive control processes (such as working memory, decision making and inhibition) in patients during the antisaccades compared to visually guided trials which do not require these additional higher-order processes. The processing impairment may imply that both mechanisms involved in the antisaccade paradigm—inhibition of reflexive misdirected saccades and triggering of intentional correct antisaccades—may be impaired in patients.

Next, we compared controls and patients in the gap, step and overlap conditions based on the outcomes of saccade latencies and error rates. We found longer latencies in patients than in controls in all conditions. Saccadic latency reflects visual processing, target selection, and motor programming and is dependent on stimulus properties, such as luminance and the nature of the cognitive task (Leigh & Kennard, 2004). Therefore, longer latencies in patients may indicate defects in the usage and interpretation of visual information, poor selection of single object from a field of multiple objects as the goal of a movement, and defective transformation of abstract codes into spatially and temporally coordinated patterns of muscle contractions that produce EMs. In addition, a longer saccade may also reflect poor disengagement, shift, and re-engagement of visual attention.

When we compared the latencies in the gap condition to those in step and overlap conditions, we found a gap effect, manifested by a significant reduction in prosaccade latency in the gap condition compared with the overlap and step conditions. The gap paradigm elicited shorter latency saccades than the step and overlap conditions in both patients and controls. The gap latencies are generally shorter than in other conditions because the gap stimuli primarily release the eye fixation mechanism for a change in gaze direction and provide a warning cue when the fixation stimulus is offset. There is a drop in fixation neuronal discharge approximately 100 msec into the gap period and a slow buildup of low-frequency activity among a subset of saccade neurons in both the SC and FEF (Dias & Bruce, 1994; Dorris & Munoz, 1995; Dorris et al., 1997; Everling & Munoz, 2000; D. P. Munoz & Wurtz, 1995). The rostral pole of the midbrain superior colliculus, which projects to omnipause neurons, plays an important role in the release of fixation and warning components. When we compared the gap effect size in patients and controls, we found a seemingly large effect in both groups, although the mean magnitude of the effect was larger in controls (1.25 in controls vs. 1.23 in patients). However, further quantitative analyses to investigate the gap effect significance between controls and patients were not feasible since the variance (SD) in the difference for latency between gap and overlap conditions from individual studies could not be obtained. These findings may suggest differences in the neuronal activity of the fixation neurons and saccade neurons during the gap period, with patients having a slower decline in fixation neuronal activity and/or a slower buildup of saccade neuronal activity. This substantiates previous findings in the literature that compared younger and older adults and found that the absolute size of the gap effect varied between age groups, but the relative decrease in latency remained constant (Pratt et al., 1997). Crawford et al. (2013) found that the size of the gap effect did not differ significantly when older controls were compared to patients with ADD, but it was significantly different in younger controls (Crawford et al., 2013).

When we compared the latencies in the visually guided saccades to the antisaccade tasks (i.e., anti-effect), the meta-analysis results showed a large effect size manifested by significantly longer latencies in antisaccade than in prosaccade tasks in both controls and patients. The longer reaction time in antisaccade reflects additional processing and higher-order cognitive control processes during the antisaccades. However, further quantitative analyses to further investigate the anti-effect significance between controls and patients were not feasible since the variance (SD) of the difference for saccade conditions latency between visually guided and antisaccade paradigms from individual studies could not be obtained.

Furthermore, we found more antisaccade errors in patients than in controls, suggesting that patients are unable to inhibit reflex saccades, possibly due to DLPFC and ACC lesions and insufficient top-down inhibition of saccade neurons in the FEF and SC before the target appearance (Douglas P. Munoz & Everling, 2004).

Finally, in the prosaccade paradigm, gap and overlap conditions may be able to distinguish MCI from ADD using latency as an outcome as we found a medium effect size in the ADD group and a small effect size in the MCI group, with no overall difference between patients with MCI and healthy controls. Similarly, in the antisaccade paradigm with gap condition, the frequency of errors revealed a difference between patients with MCI and patients with ADD when both groups were compared with controls, and we found a large effect size in the ADD group and medium effects in the MCI group, with no overall difference between patients with MCI and controls. Patients with MCI are presumed to have better performance than patients with ADD in tasks related to increased cognitive load, visual attention, disengagement and attention shift as there were not many significant differences when they were compared with controls. When we compared directly the ADD groups with MCI groups, in the gap condition, we found an overall medium effect size in prosaccade latency with statistical significance, and small to medium effect sizes in the antisaccade latency and error rate (with marginal CIs). Similarly, medium effect size (with marginal CI) was observed in the prosaccade latency in the overlap condition. In these direct comparisons between ADD and MCI, we have limited number of studies (4 to 5) and more studies are required to confirm the results.

### Limitations and Future Directions

This review has several limitations. First, our results were derived from observational study designs that are prone to several limitations, mainly due to unmeasured confounding factors and other risks of bias. We used the risk of bias assessment as a measure of quantification to limit bias in the final inclusion. Additionally, our primary analysis was based on the differentiation of participants in terms of the saccade task condition. This limited the number of comparisons when the studies selected had a small number of manipulations, such as step conditions.

Given that the analysis focused on horizontal saccades, there is some likelihood that dissimilar evaluations would have arisen if the focus was on vertical saccades. This is because horizontal and vertical saccades are generated by distinct groups of premotor neurons (Leigh & Kennard, 2004; Takahashi & Shinoda, 2018). Additionally, several studies had either controls or patients with MCI and ADD but not both patient groups; therefore, we were unable to carry out subgroup analyses.

In addition, there was a lack of adequate information or discrepancies in the categorization of saccades (such as anticipatory and predictive) by different studies, which may have impacted the saccade parameter results reported. Some studies had a specific criterion of saccades that clearly differentiated the different saccade behaviors, such as anticipatory and express saccades. The range of saccade behaviors, such as memory-guided, predictive, and reflexive saccades, could not be explored in depth (Leigh & Kennard, 2004). Additionally, antisaccade metrics such as error rates, correct antisaccade latency and error latency were not defined in all studies. Therefore, because it was likely that studies described the measures differently due to lack of agreement in definitions of saccadic parameters, it was impractical to determine precise differences between controls and patients.

When extracting data, we relied on data extraction software such as WebPlot digitizer, whose accuracy is dependent on the quality of images (provided) in the manuscript and may therefore be prone to variation from the actual results. In addition, it was not possible to investigate the significant relationships of controls and patients further because the variance (standard deviation) of the difference for the anti-effect, gap effect, and saccade conditions could not be obtained from the studies.

We conducted a broad search of several databases but placed restrictions on the language of the study. Only studies published in English were considered in this review, which is one of the main limitations. It is likely that there are other studies published in other languages that we have missed in this review.

Another potential limitation of this review is the possibility of publication bias. Overall, many studies retrieved and included in the review reported statistical comparisons between controls and patients in the gap and overlap condition that did not reach significance. Generally, the best way to minimize the impact of publication bias in a systematic review is the inclusion of trial registries and unpublished studies or grey literature (Lau et al., 2006; Sterne et al., 2011). Since we included only published articles, there is a high chance that several other completed studies may not have been published due to inconclusive results. Other than publication bias, reasons that may explain the funnel plot asymmetry include poor methodological quality leading to exaggerated effects in smaller studies, true variation across studies, artefactual causes and chance (Sterne et al., 2011).

Since the focus of the study was on MCI caused by any etiology, there is a possibility that dissimilar evaluations would have arisen if the focus had been on MCI due to AD.

Finally, we mostly examined studies that used gap and overlap stimulus paradigms to test saccades. We mainly used latency in the gap and overlap conditions for calculating the mean differences because it was the most common measure in the studies. Other saccade parameters, such as amplitude, gain, and velocity, need to be investigated to determine whether there are significant differences between controls and patients. Future studies should explore step, different ranges of saccade behaviors (such as anticipations, reflexive, express), smooth pursuit, mixed tasks, saccade parameters, such as peak velocity, amplitude, and fixation, and other neurological or psychiatric pathologies that affect saccades. Additionally, visually guided eye movements were shown to be prone to disease, ageing and ethnicity (Polden et al., 2020). Therefore, future research should explore saccade performance based on these variables.

## Conclusion

The main goal of the current study was to determine whether different saccade paradigms and conditions could distinguish patients with MCI and patients with ADD from controls and validate the gap effect and anti-effect in patients with MCI and ADD compared to controls. We found that, in general, patients can be distinguished from controls by prosaccade and antisaccade latencies and frequency of antisaccade errors, regardless of the saccade condition. Both prosaccade and antisaccade paradigms differentiated patients from controls. More specifically, antisaccade paradigms were more effective than prosaccade paradigms in distinguishing patients from controls, as shown by a large effect size in antisaccade paradigms and moderate effect in prosaccade paradigms. During prosaccades in the gap and overlap conditions, when patients were compared with controls, patients with ADD had significantly longer latencies than patients with MCI, and these latencies, corresponding to a moderate effect size in ADD and a small effect size in MCI, could be used to differentiate the two groups. Similarly, during antisaccades in the gap condition, when patients were compared with controls, patients with ADD had significantly more errors than patients with MCI, and these errors, corresponding to a large effect size in ADD and a moderate effect size in MCI, could be used to differentiate the two groups. The absolute size of the gap effect varied between participant groups, but the relative decrease in latency remained constant, with both groups showing a large effect size. The anti-effect magnitude was similar in both patients and controls; however, patients with MCI had longer antisaccade latencies than patients with ADD, corresponding to a moderate effect size in ADD and a large effect size in MCI. In conclusion, the results offer compelling evidence supporting the use of gap effect, anti-effect and specific saccade paradigms and conditions to distinguish between MCI and ADD patients as well as between patients and controls.

## Supplementary Information

Below is the link to the electronic supplementary material.

Supplementary file1 (DOCX 8937 KB)
