# Supplementary material for: Saccadic Eye Movement in Mild Cognitive Impairment and Alzheimer’s Disease: A Systematic Review and Meta-Analysis
Source: Neuropsychol Rev. 2021 May 6;32(2):193–227. doi: 10.1007/s11065-021-09495-3 (PMC9090874; doi:10.1007/s11065-021-09495-3)
Supplement: Supplementary file 1 — Supplementary file1 (DOCX 8937 KB) [file 11065_2021_9495_MOESM1_ESM.docx]

**Supplementary Material**

**Saccadic Eye Movement in Mild Cognitive Impairment and Alzheimer’s Disease: a Systematic Review and Meta-Analysis**

**Table S1**: PubMed MEDLINE search strategy

| **Sequence** | **Keyword(s) and Field** |
| --- | --- |
| 1 | "Alzheimer Disease"[Mesh] |
| 2 | alzheimer*[Title/Abstract] |
| 3 | AD[Title/Abstract] |
| 4 | "Dementia, multi-Infarct"[Mesh] |
| 5 | MCI[Title/Abstract] |
| 6 | amnestic[Title/Abstract] |
| 7 | (cognit* impair*)[Title/Abstract] |
| 8 | (cognit* declin*)[Title/Abstract] |
| 9 | (cognit* deficit*)[Title/Abstract] |
| 10 | (cognit* disturb*)[Title/Abstract] |
| 11 | “cognitive disorders”[Title/Abstract] |
| 12 | (cognit* defect*)[Title/Abstract] |
| 13 | "Cognitive defect"[Title/Abstract] |
| 14 | "Cognition Disorders"[Mesh] |
| 15 | "Amnesia"[Mesh] |
| 16 | "memory impairment"[Title/Abstract] |
| 17 | CDR AND 0.5[Title/Abstract] |
| 18 | "clinical dementia rating" AND 0.5[Title/Abstract] |
| 19 | dement*[Title/Abstract] |
| 20 | "mild neurocognitive disorder"[Title/Abstract] |
| 21 | "Subjective memory complaints"[Title/Abstract] OR SMC[Title/Abstract] |
| 22 | OR 1-21 |
| 23 | “electrooculography”[Mesh] |
| 24 | “electrooculograph”[Title/Abstract] |
| 25 | “EOG”[Title/Abstract] |
| 26 | “eye movements”[Mesh] |
| 27 | “eye movement”[Title/Abstract] |
| 28 | (videooculograph*)[ti/ab] OR “VOG”[ti/ab] |
| 29 | “eye tracking”[ti/ab] |
| 30 | “gaze tracking”[ti/ab] |
| 31 | “scleral contact lens”[ti/ab] |
| 32 | “pupil reflection” [ti/ab] |
| 33 | “corneal reflection” [ti/ab] |
| 34 | “search coil” [ti/ab] |
| 35 | OR 23-34 |
| 36 | #22 AND #35 |

**Table S2**: EMBASE search strategy

| **Sequence** | **Keyword(s) and Field** |
| --- | --- |
| 1 | 'alzheimer disease'/exp |
| 2 | alzheimer*:ab,ti |
| 3 | AD:ab,ti |
| 4 | 'multiinfarct dementia'/exp |
| 5 | MCI:ab,ti |
| 6 | amnestic:ab,ti |
| 7 | ‘cognit* impair*’:ab,ti |
| 8 | ‘cognit* declin*’:ab,ti |
| 9 | ‘cognit* deficit*’:ab,ti |
| 10 | 'cognit* disturb*':ab,ti |
| 11 | ‘cognit* defect*’:ab,ti |
| 12 | 'cognitive defect'/exp |
| 13 | 'memory disorder'/exp |
| 14 | 'cognitive disorders'/exp |
| 15 | ‘anterograde amnesia'/exp |
| 16 | ‘retrograde amnesia'/exp |
| 17 | ‘transient global amnesia'/exp |
| 18 | 'memory impairment':ab,ti |
| 19 | (CDR NEAR/3 0.5):ab,ti OR ('clinical dementia rating' NEAR/3 0.5):ab,ti |
| 20 | dement*:ab,ti |
| 21 | 'mild neurocognit* disorder*':ab,ti |
| 22 | 'subjective memory complaints':ab,ti OR SMC:ab,ti |
| 23 | ‘electrooculography’/exp |
| 24 | electrooculograph*:ab,ti |
| 25 | ‘electrooculography’:ab,ti |
| 26 | ‘eye movements’/exp |
| 27 | ‘eye movement*’:ab,ti |
| 28 | ‘videooculograph*’:ab,ti |
| 29 | VOG:ab,ti |
| 30 | ‘eye tracking’:ab,ti |
| 31 | ‘gaze tracking’:ab,ti |
| 32 | ‘scleral contact lens’:ab,ti |
| 33 | ‘pupil reflection’:ab,ti |
| 34 | ‘corneal reflection’:ab,ti |
| 35 | ‘search coi’:ab,ti |
| 36 | OR 1-22 |
| 37 | OR 23-35 |
| 38 | #36 AND #37 |

**Table S3**: Cochrane search strategy

| **Sequence** | **Keyword(s) and Field** |
| --- | --- |
| 1 | 'alzheimer disease'/exp |
| 2 | alzheimer*:ab,ti |
| 3 | AD:ab,ti |
| 4 | 'multiinfarct dementia'/exp |
| 5 | MCI:ab,ti |
| 6 | amnestic:ab,ti |
| 7 | ‘cognit* impair*’:ab,ti |
| 8 | ‘cognit* declin*’:ab,ti |
| 9 | ‘cognit* deficit*’:ab,ti |
| 10 | 'cognit* disturb*':ab,ti |
| 11 | ‘cognit* defect*’:ab,ti |
| 12 | 'cognitive defect'/exp |
| 13 | 'memory disorder'/exp |
| 14 | 'cognitive disorders'/exp |
| 15 | ‘anterograde amnesia'/exp |
| 16 | ‘retrograde amnesia'/exp |
| 17 | ‘transient global amnesia'/exp |
| 18 | 'memory impairment':ab,ti |
| 19 | (CDR NEAR/3 0.5):ab,ti OR ('clinical dementia rating' NEAR/3 0.5):ab,ti |
| 20 | dement*:ab,ti |
| 21 | 'mild neurocognit* disorder*':ab,ti |
| 22 | 'subjective memory complaints':ab,ti OR SMC:ab,ti |
| 23 | ‘electrooculography’/exp |
| 24 | electrooculograph*:ab,ti |
| 25 | ‘electrooculography’:ab,ti |
| 26 | ‘eye movements’/exp |
| 27 | ‘eye movement*’:ab,ti |
| 28 | ‘videooculograph*’:ab,ti |
| 29 | VOG:ab,ti |
| 30 | ‘eye tracking’:ab,ti |
| 31 | ‘gaze tracking’:ab,ti |
| 32 | ‘scleral contact lens’:ab,ti |
| 33 | ‘pupil reflection’:ab,ti |
| 34 | ‘corneal reflection’:ab,ti |
| 35 | ‘search coi’:ab,ti |
| 36 | OR 1-22 |
| 37 | OR 23-35 |
| 38 | #36 AND #37 |

**Table S4**: Risk Of Bias In Non-randomized Studies of Interventions (ROBINS-I) assessment

| Risk Of Bias In Non-randomized Studies of Interventions (ROBINS-I) assessment | | | | |
| --- | --- | --- | --- | --- |
| No | Title | First Author | Year of Publication | Overall Bias |
| 1 | A Spatial Decision Eye-Tracking Task in Patients with Prodromal and Mild Alzheimer's Disease | Laurens, B. | 2019 | Moderate |
| 2 | Abnormalities of fixation, saccade and pursuit in posterior cortical atrophy | Shakespeare, T. J. | 2015 | Moderate |
| 3 | Abnormalities of saccadic eye movements in dementia due to Alzheimer’s disease and mild cognitive impairment | Wilcockson, T. D. W. | 2019 | Moderate |
| 4 | Animal spotting in Alzheimer's disease: an eye tracking study of object categorization | Boucart, M. | 2014 | Low |
| 5 | Antisaccade task reflects cortical involvement in mild cognitive impairment | Heuer, H. W. | 2013 | Low |
| 6 | Attentional capture by incongruent object/background scenes in patients with Alzheimer’s disease: A saccadic choice task | Lenoble, Q. | 2018 | Moderate |
| 7 | Behavioral and cognitive markers of mild cognitive impairment: diagnostic value of saccadic eye movements and Simon task | Chehrehnegar, N. | 2019 | Moderate |
| 8 | Changes in visual fixation and saccadic eye movements in Alzheimer's disease | Bylsma, F. W. | 1995 | Moderate |
| 9 | Differential processing of natural scenes in posterior cortical atrophy and in Alzheimer's disease, as measured with a saccade choice task | Boucart, M. | 2014 | Moderate |
| 10 | Early Emotional Attention is Impacted in Alzheimer's Disease: An Eye-Tracking Study | Bourgin, J. | 2018 | Moderate |
| 11 | Effects of stimulus predictability and inter-stimulus gap on saccades in Alzheimer's disease | Abel, L. A. | 2002 | Moderate |
| 12 | Executive deficits detected in mild Alzheimer's disease using the antisaccade task | Kaufman, L. D. | 2012 | Moderate |
| 13 | Eye-tracking metrics in young onset Alzheimer's disease: A Window into cognitive visual functions | Pavisic, I. M. | 2017 | Low |
| 14 | Impairment of spatially directed attention in patients with probable Alzheimer's disease as measured by eye movements | Scinto, L. F. | 1994 | Moderate |
| 15 | Long Latency and High Variability in Accuracy-Speed of Prosaccades in Alzheimer's Disease at Mild to Moderate Stage | Yang, Q. | 2011 | Moderate |
| 16 | Medial versus lateral frontal lobe contributions to voluntary saccade control as revealed by the study of patients with frontal lobe degeneration | Boxer, A. L. | 2006 | Moderate |
| 17 | Neural correlates of saccadic inhibition in healthy elderly and patients with amnestic mild cognitive impairment | Alichniewicz, K. K. | 2013 | Moderate |
| 18 | Oculomotor function in frontotemporal lobar degeneration, related disorders and Alzheimer's disease | Garbutt, S. | 2008 | Low |
| 19 | Prodromal Alzheimer's Disease Demonstrates Increased Errors at a Simple and Automated Anti-Saccade Task | Holden, J. G. | 2018 | Moderate |
| 20 | Saccade abnormalities in autopsy-confirmed frontotemporal lobar degeneration and Alzheimer disease | Boxer, A. L. | 2012 | Low |
| 21 | Saccade deficits in amnestic mild cognitive impairment resemble mild Alzheimer's disease | Peltsch, A. | 2014 | Moderate |
| 22 | Saccadic eye movement changes in Parkinson's disease dementia and dementia with Lewy bodies | Mosimann, U. P. | 2005 | Moderate |
| 23 | Saccadic Eye Movements and Attentional Control in Alzheimer's Disease | Noiret, N. | 2018 | Low |
| 24 | Saccadic latency measurements in dementia | Hershey, L. A. | 1983 | Moderate |
| 25 | Scene categorization in Alzheimer's disease: a saccadic choice task | Lenoble Q. | 2015 | Low |
| 26 | Specific saccade deficits in patients with Alzheimer’s disease at mild to moderate stage and in patients with amnestic mild cognitive impairment | Yang, Q. | 2013 | Low |
| 27 | Spectrum of saccade system function in Alzheimer disease | Shafiq-Antonacci, R. | 2003 | Moderate |
| 28 | The disengagement of visual attention in Alzheimer's disease: a longitudinal eye-tracking study | Crawford, T. J. | 2015 | Moderate |
| 29 | The effect of neurodegeneration on visuomotor behavior in Alzheimer's disease and Parkinson's disease | De Boer, C. | 2016 | Moderate |
| 30 | The Effects of Previous Error and Success in Alzheimer’s Disease and Mild Cognitive Impairment | Crawford, T. J.2019 | 2019 | Moderate |
| 31 | The role of working memory and attentional disengagement on inhibitory control: effects of aging and Alzheimer's disease | Crawford, T. J. | 2013 | Low |
| 32 | Validation of a clinical anti-saccadic eye movement test in the assessment of dementia | Currie, J. | 1991 | Moderate |
| 33 | Visual exploration behaviour during clock reading in Alzheimer’s disease | Mosimann, U. P. | 2004 | Moderate |
| 34 | Visuomotor impairment in early-stage Alzheimer's disease: Changes in relative timing of eye and hand movements | Verheij, S. | 2012 | Moderate |
| 35 | The Disengagement of Visual Attention:An Eye-Tracking Study of Cognitive Impairment,Ethnicity and Age | Polden,M. | 2020 | Low |

**Forest plots**

**A. Gap**
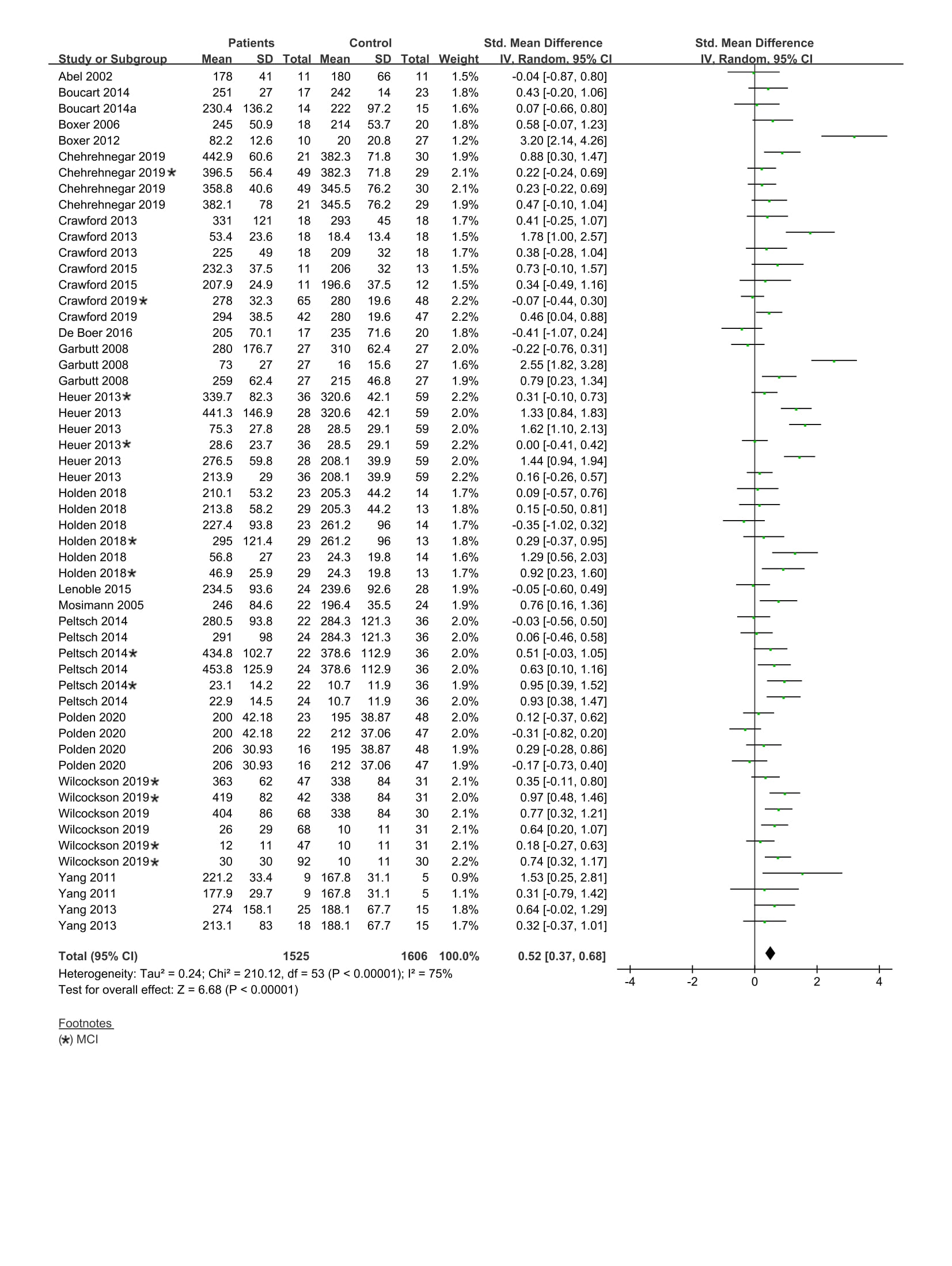


**Fig. A1** Forest plot of effect sizes and their confidence intervals, comparing patients and controls in the gap condition. Std. Mean Difference = Standardized mean differences and expressed as Hedges’ g (unbiased).


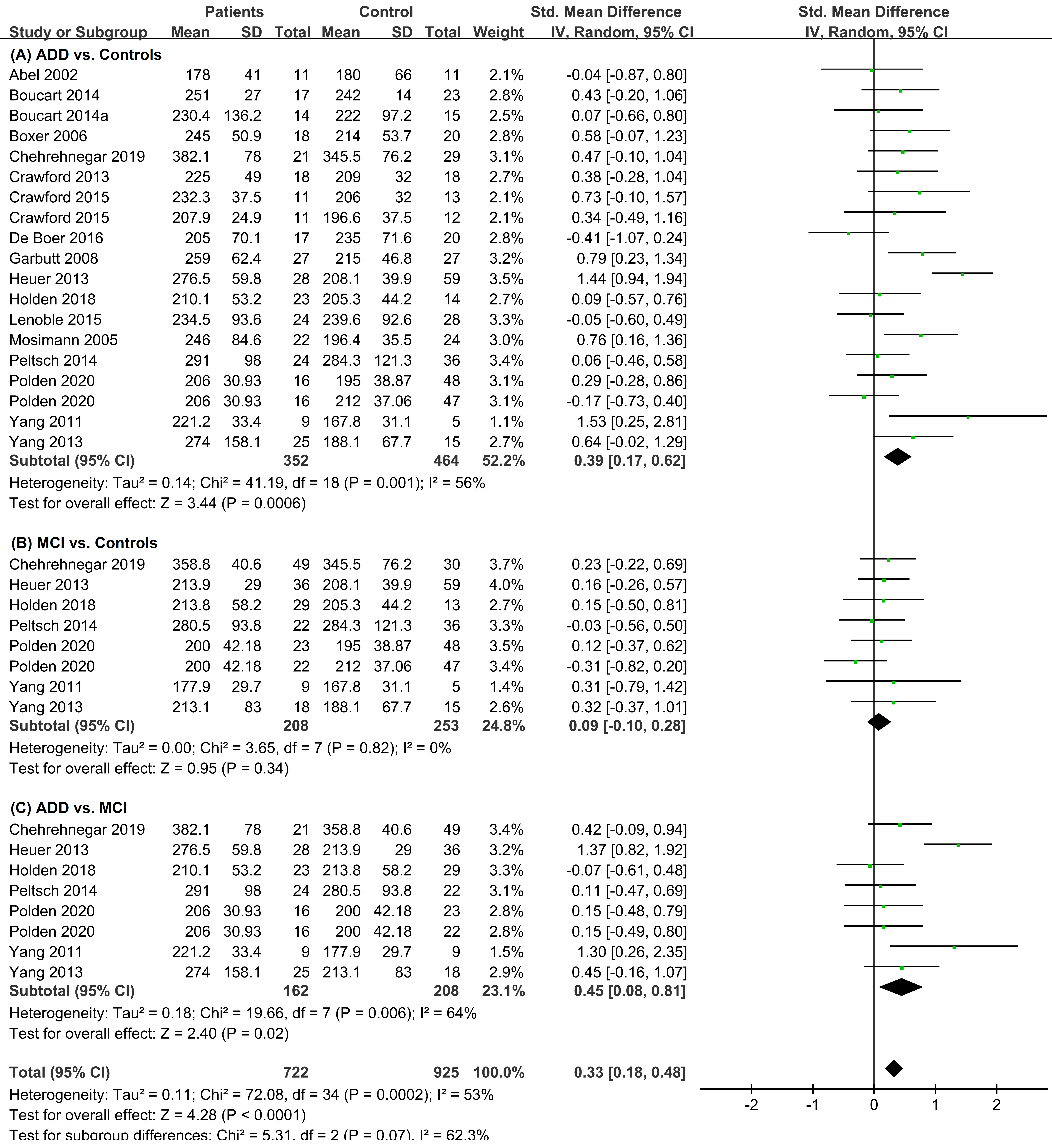


**Fig. A2** Forest plot of effect sizes and their confidence intervals, comparing prosaccade latency (msec) in the gap condition for (A) patients with ADD and controls, (B) patients with MCI and controls, and (C) patients with ADD and patients with MCI. Std. Mean Difference = Standardized mean differences and expressed as Hedges’ g (unbiased).


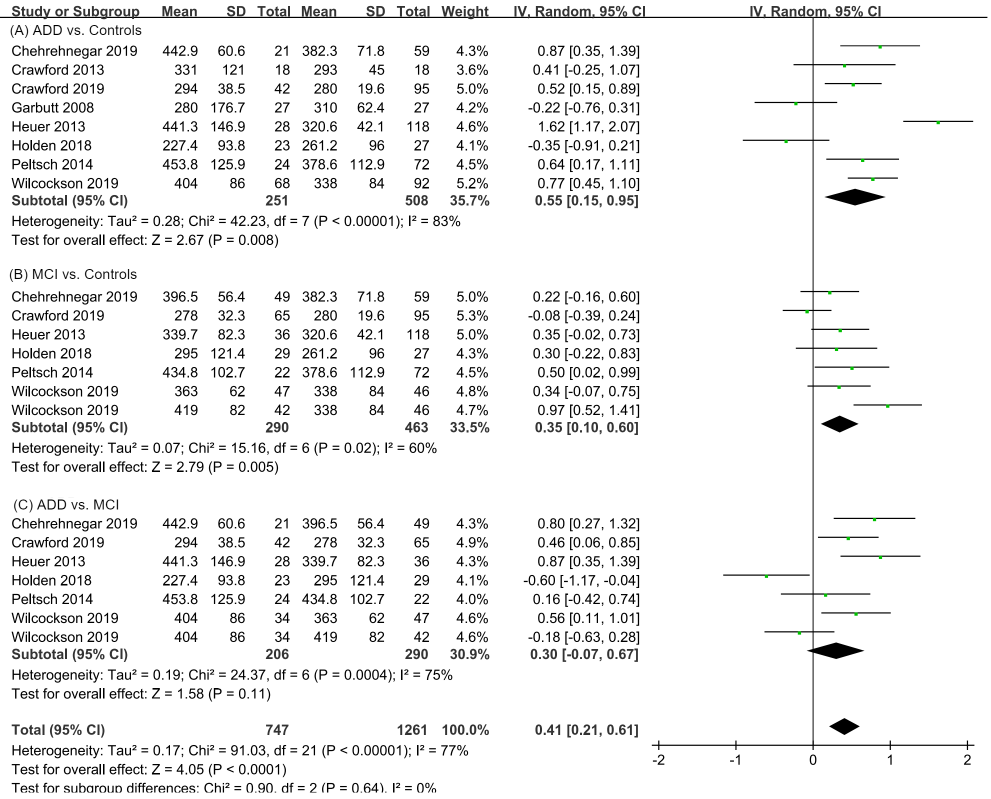


**Fig. A3** Forest plot of effect sizes and their confidence intervals, comparing antisaccade latency (msec) in the gap condition for (A) patients with ADD and controls, (B) patients with MCI and controls, and (C) patients with ADD and patients with MCI. Std. Mean Difference = Standardized mean differences and expressed as Hedges’ g (unbiased).


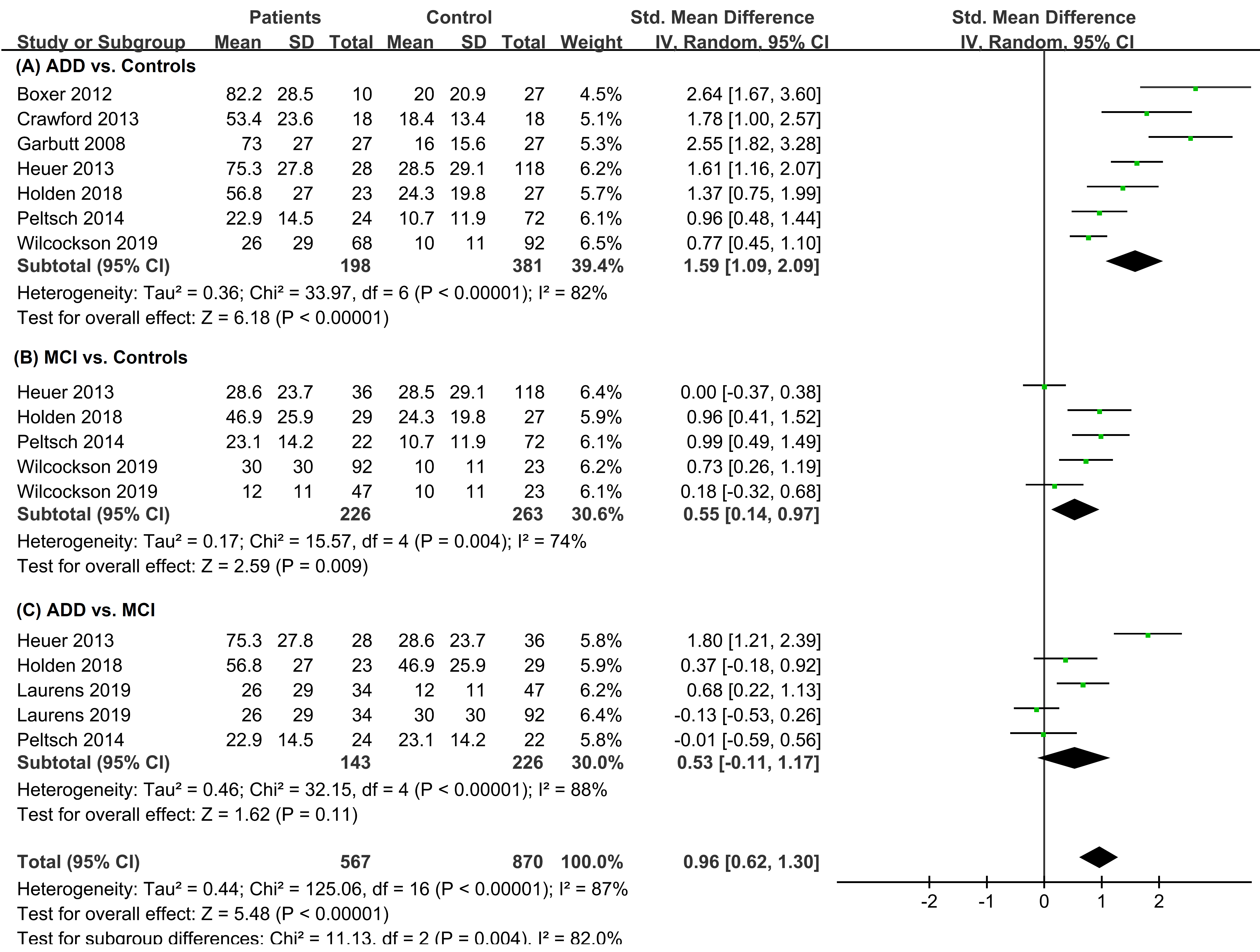
 **Fig. A4** Forest plot of effect sizes and their confidence intervals, comparing antisaccade error rate (%) in the gap condition for (A) patients with ADD and controls, (B) patients with MCI and controls, and (C) patients with ADD and patients with MCI. Std. Mean Difference = Standardized mean differences and expressed as Hedges’ g (unbiased).

**B. Step**
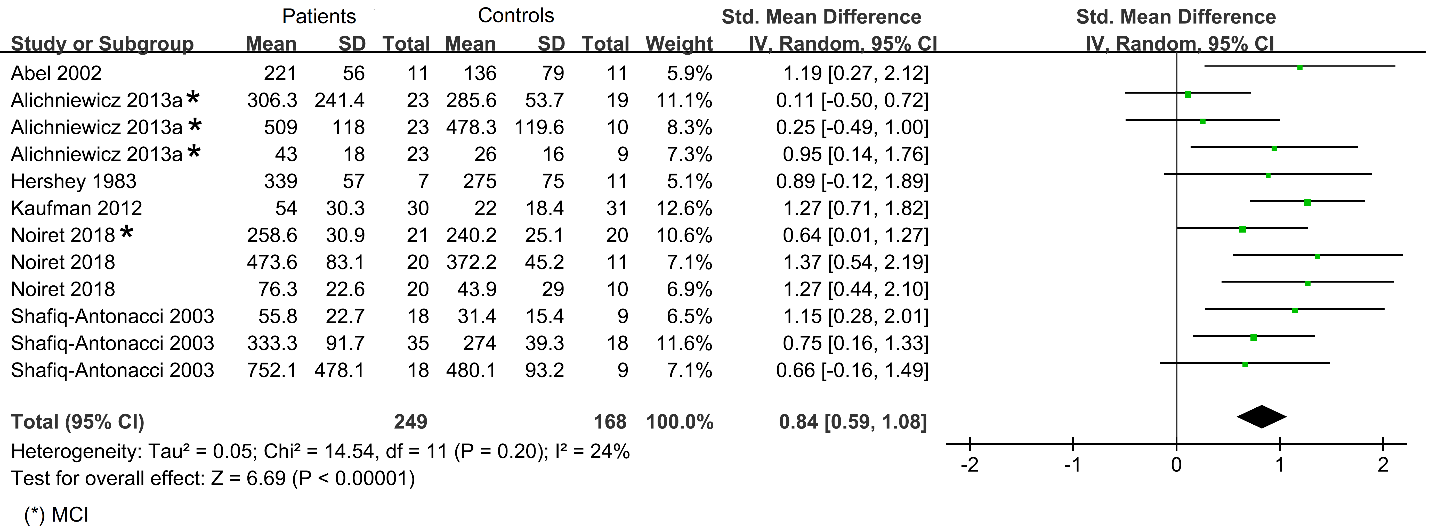


**Fig. B1** Forest plot of effect sizes and their confidence intervals, comparing patients and controls in the step condition. Std. Mean Difference = Standardized mean differences and expressed as Hedges’ g (unbiased).

**C. Overlap**
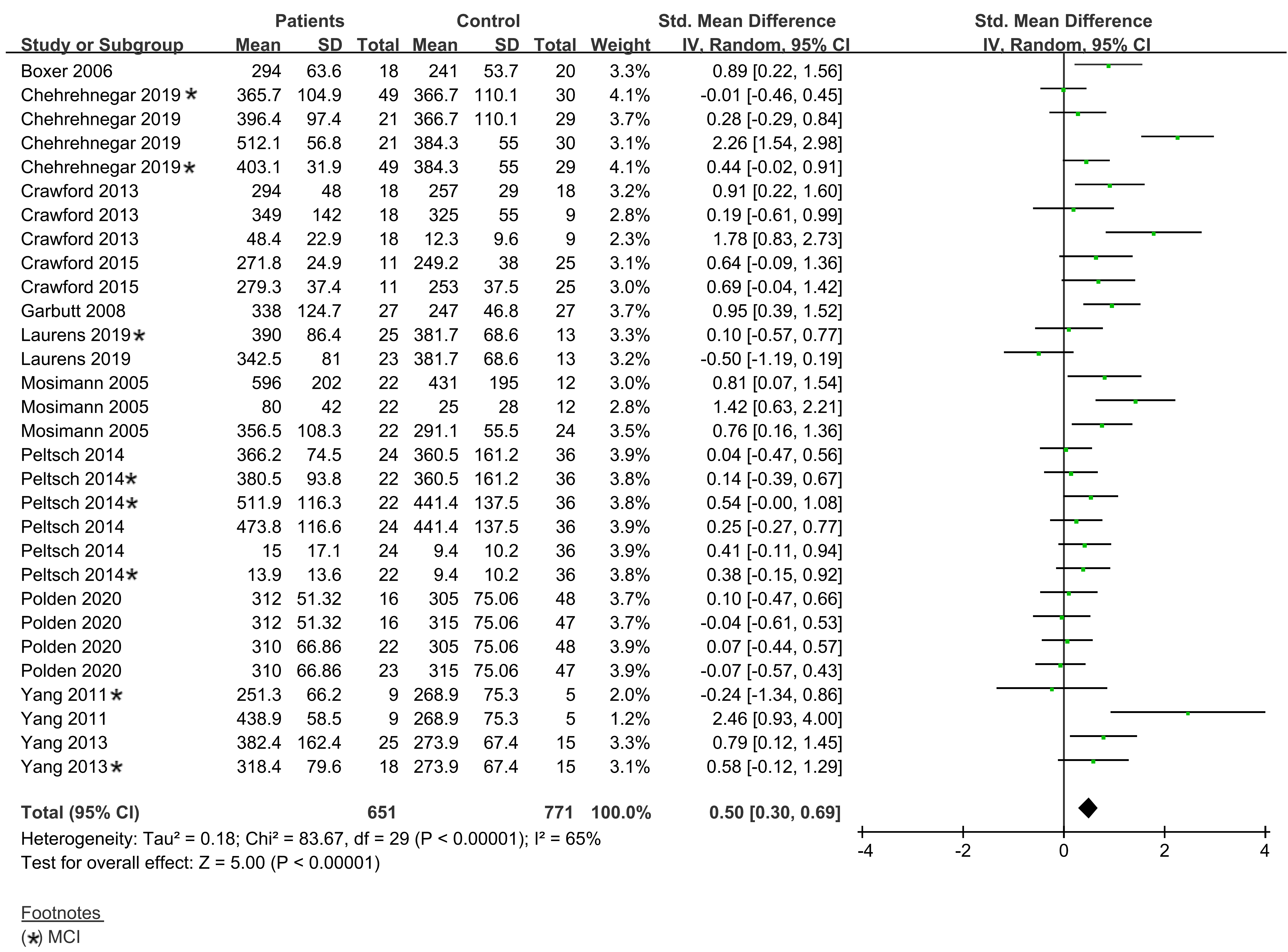
**Fig. C1** Forest plot of effect sizes and their confidence intervals, comparing patients and controls in the overlap condition. Std. Mean Difference = Standardized mean differences and expressed as Hedges’ g (unbiased).


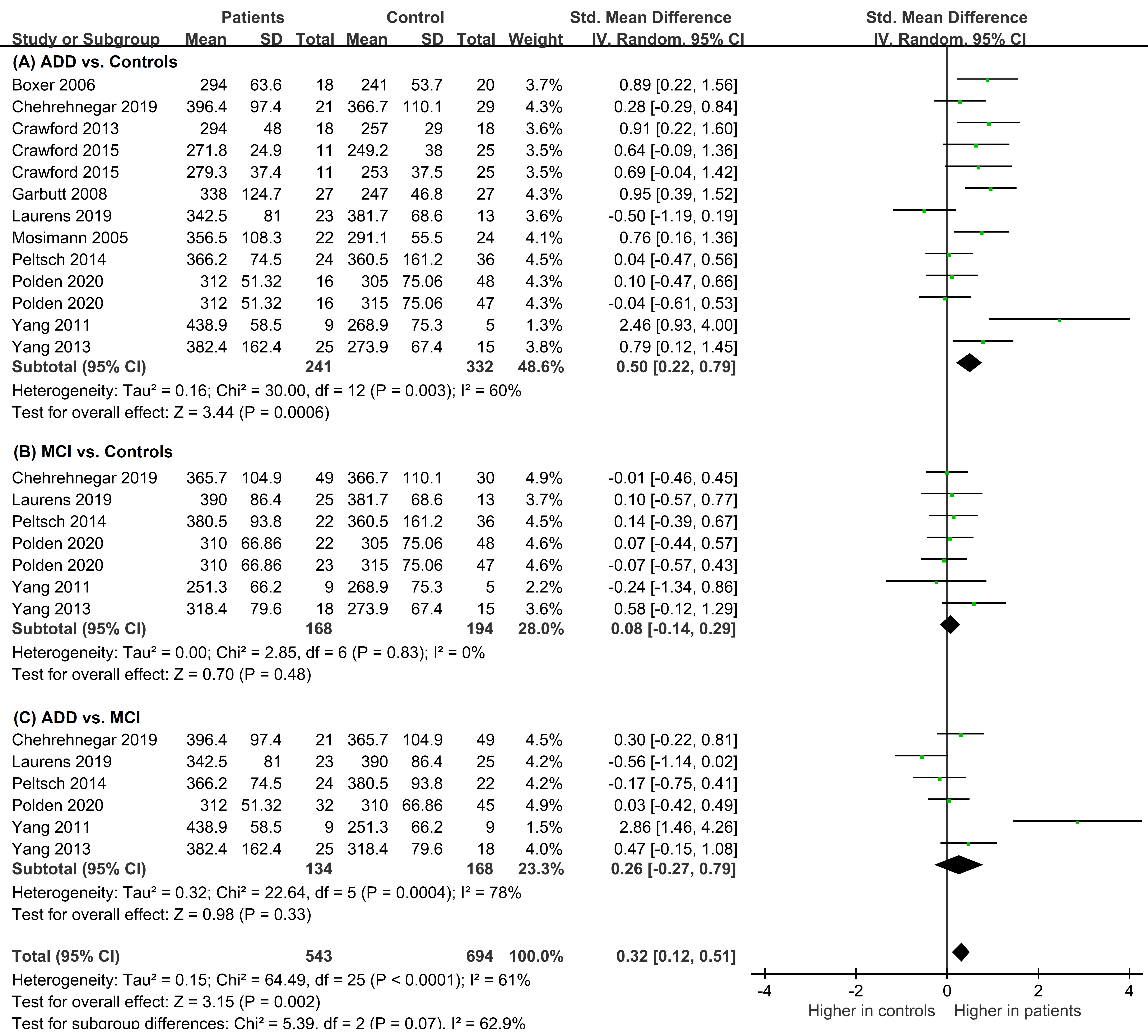
**Fig. C2** Forest plot of effect sizes and their confidence intervals, comparing prosaccade latency (msec) in the overlap condition for (A) patients with ADD and controls, (B) patients with MCI and controls, and (C) patients with ADD and patients with MCI. Std. Mean Difference = Standardized mean differences and expressed as Hedges’ g (unbiased).

**D. Gap-effect**
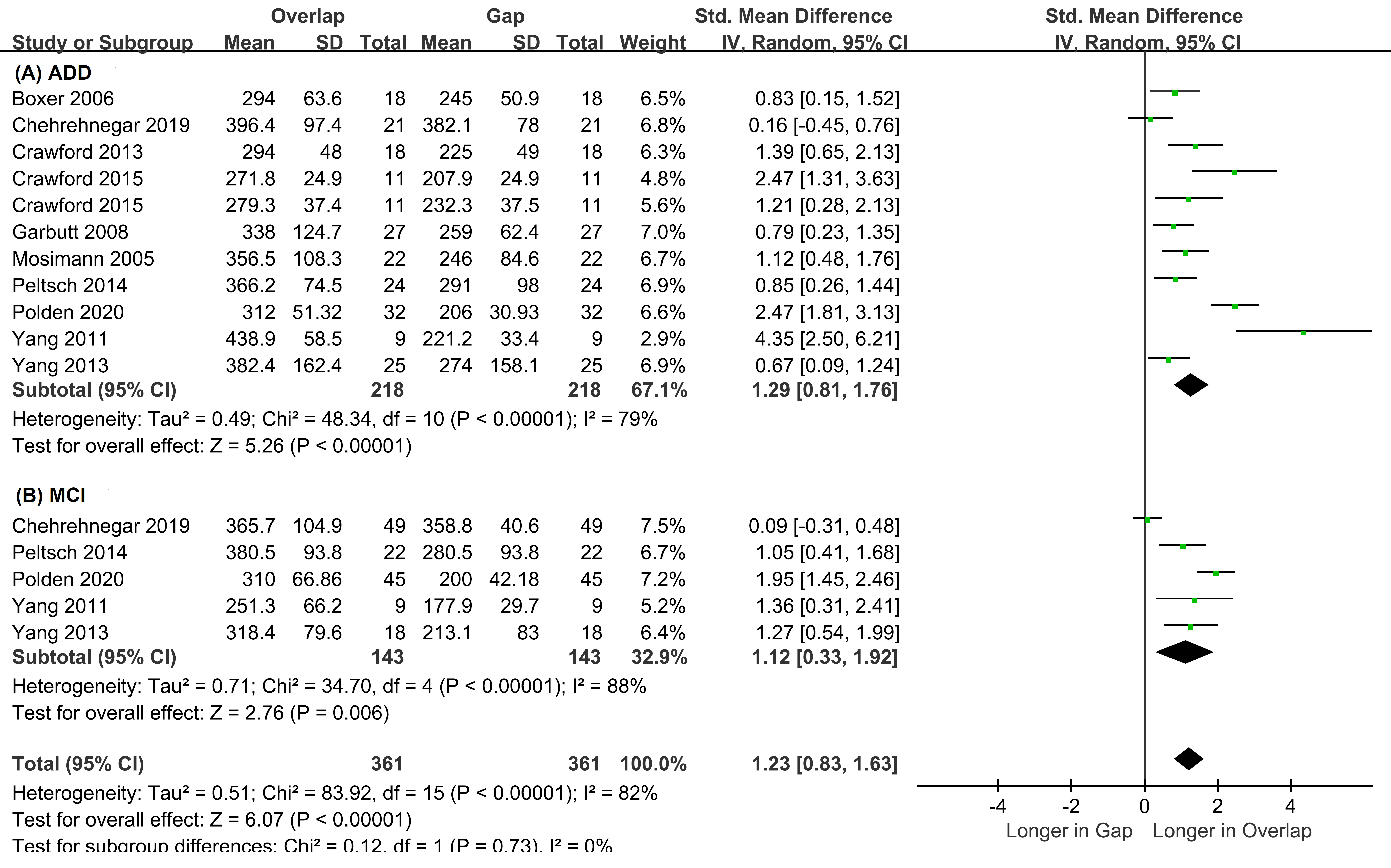
**Fig. D1** Forest plot of effect sizes and their confidence intervals, comparing prosaccade latency (msec) between overlap and gap conditions for (A) patients with ADD, and (B) patients with MCI

**E. Anti-effect**
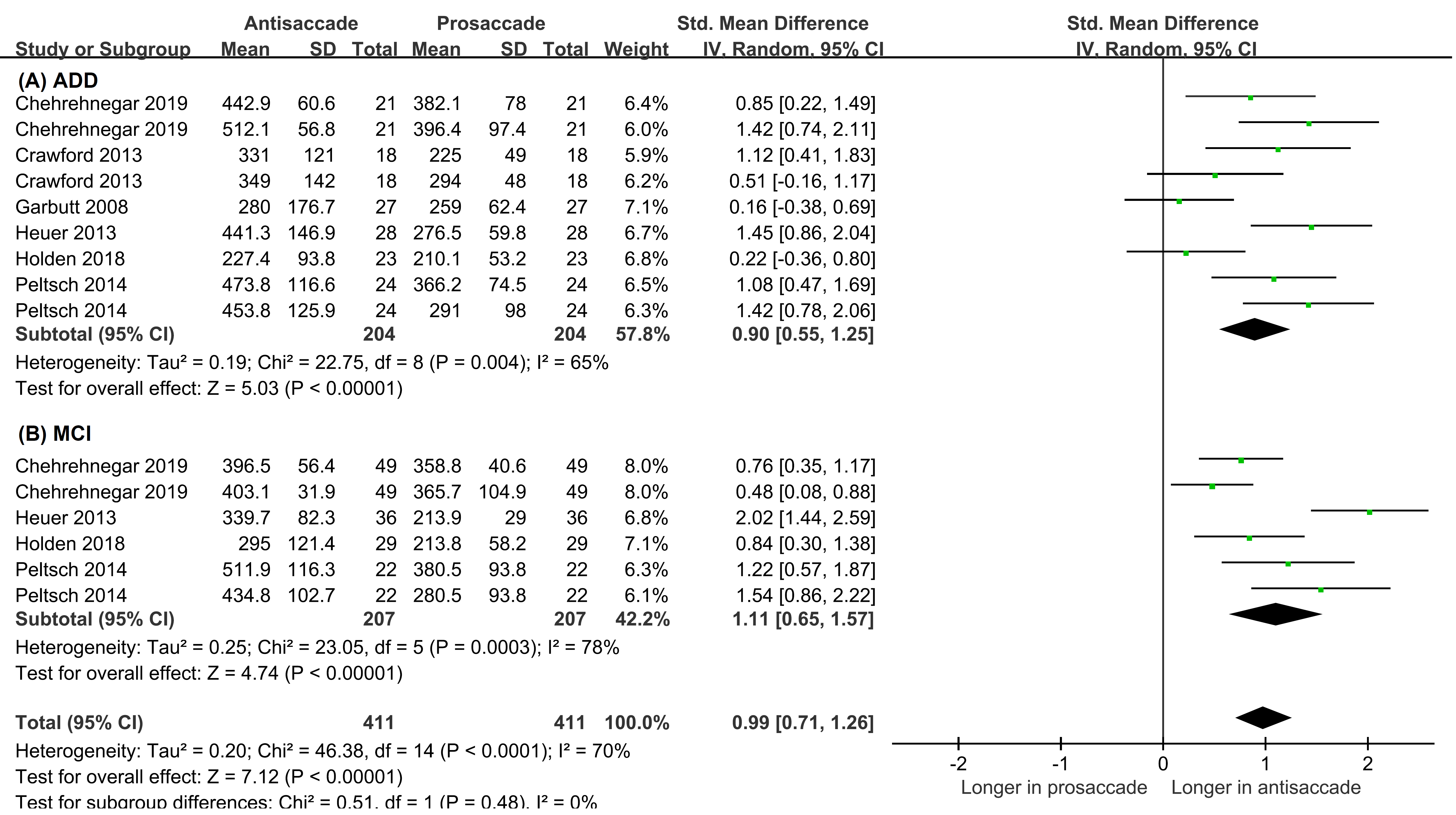
**Fig. E1** Forest plot of effect sizes and their confidence interval, comparing latencies (msec) between antisaccade and prosaccade in gap and overlap conditions for (A) patients with ADD, and (B) patients with MCI.
